# Pemafibrate modulates peroxisome proliferator-activated receptor alpha and prevents alcohol-associated liver disease in rats

**DOI:** 10.1186/s10020-025-01210-9

**Published:** 2025-04-22

**Authors:** Takashi Saito, Joseph George, Kazuaki Ozaki, Mutsumi Tsuchishima, Mikihiro Tsutsumi

**Affiliations:** 1 Department of Hepatology, Kanazawa Medical University, Uchinada, Ishikawa 920-0293 Japan; 2 Center for Regenerative Medicine, Kanazawa Medical University Hospital, Uchinada, Ishikawa 920-0293 Japan

**Keywords:** Alcohol-associated liver disease, Steatosis, Steatohepatitis, PPARα, Pemafibrate

## Abstract

**Background and aims:**

Alcohol-associated liver disease (ALD) with steatosis or steatohepatitis that could progress to liver cirrhosis is a common problem in chronic alcohol consumption. Pemafibrate is a novel, highly specific peroxisome proliferator-activated receptor-α (PPARα) modulator, which regulates the expression of the target genes related to lipid and glucose metabolism. Here, we evaluated the effect of pemafibrate to prevent ALD and steatosis in rats.

**Methods:**

The animals were treated with liquid diet containing ethanol (36% of total calories) or an isocaloric carbohydrate diet for 4 weeks. Subsequently, both groups were fed with either 0.5% aqueous methylcellulose solution (MC) or MC containing 0.3 mg/kg body weight of pemafibrate orally twice a day along with the liquid diet for another 4 weeks. A set of animals were sacrificed at the 4th week before the start of pemafibrate treatment and the remaining animals at the end of 8 weeks. Blood and liver samples were collected for biochemical and histopathological evaluations.

**Results:**

Treatment with pemafibrate prevented inflammation and steatosis in the hepatic tissue. Furthermore, pemafibrate administration markedly increased hepatic NAD and NADH levels, reduced both serum and hepatic triglyceride levels, and upregulated the expression of molecules involved in lipid metabolism.

**Conclusions:**

The results of the present study demonstrated that pemafibrate modulates target genes related to hepatic lipid metabolism and prevents deposition of fat globules in the liver during chronic alcohol feeding in rats. Therefore, pemafibrate could be used as a potent therapeutic agent to prevent steatosis and related adverse events in ALD.

**Supplementary Information:**

The online version contains supplementary material available at 10.1186/s10020-025-01210-9.

## Highlights

- Evaluate the effects of pemafibrate to prevent alcohol-associated liver disease and steatosis.
- Pemafibrate upregulated target genes related to lipid metabolism and prevented hepatic steatosis during chronic feeding of alcohol in rats.
- Pemafibrate may be used as a potent therapeutic agent to prevent steatosis and accompanied events in alcohol-associated liver disease.

**Supplementary Information:**

The online version contains supplementary material available at 10.1186/s10020-025-01210-9.

## Introduction

Chronic alcohol consumption (alcoholism) could lead to multiple health issues that affect vital organs, leading to debilitating and life threatening conditions and death. Alcohol-associated liver disease (ALD) with steatohepatitis that could progress to liver cirrhosis and hepatocellular carcinoma is a common problem with alcoholism (Osna et al. 2017; Mackowiak et al. 2024). More than 90–95% of the patients with ALD have hepatic steatosis, 20–40% have the more advanced form, alcohol-associated steatohepatitis (ASH) with or without fibrosis, and 8–20% have liver cirrhosis (Narro et al. 2024; Mitra et al. 2020). Impaired β-oxidation of fatty acids in mitochondria is one of the major factors that contribute to hepatic steatosis during the pathogenesis of ALD (Lu and George 2024; Prasun et al. 2021). Ethanol is metabolized into acetaldehyde mainly in the cytosol by alcohol dehydrogenase (ADH), and acetaldehyde is metabolized into acetate mostly in mitochondria by aldehyde dehydrogenase (ALDH). Both ADH and ALDH require NAD^+^ as the electron acceptor with the formation of NADH (Lieber 2005). Chronic consumption of alcohol leads to disturbances in redox homeostasis and decreased activity of citric acid cycle, resulting in impaired β-oxidation of fatty acids (Harris et al. 2023). Structural and functional alterations of mitochondria due to chronic intake of alcohol may also contribute to impaired lipid metabolism in addition to the disturbances of redox homeostasis (Hoek et al. 2002).

Peroxisome proliferator-activated receptors (PPARs) are fatty acid-activated transcription factors of nuclear hormone receptor superfamily that regulate energy metabolism (Christofides et al. 2021). The three isoforms of PPARs family subtypes identified are PPARα, PPARβ/δ, and PPARγ. The major subtype, PPARα, is a ligand-activated transcription factor that mainly acts as sensors and regulators of lipid metabolism and energy homeostasis (Tyagi et al. 2011). It is highly expressed in the cells that have active fatty acid oxidation, such as hepatocytes, cardiomyocytes, enterocytes, and the proximal tubule cells of kidney (Grygiel-Górniak 2014). PPARα regulates the expression of a series of genes involved in the transport of free fatty acids, mitochondrial and peroxisomal β-oxidation, as well as ω-oxidation of fatty acids in microsomes (Lu and George 2024; Lamichane et al. 2018). Activation of PPARα promotes uptake, utilization, and catabolism of fatty acids through upregulation of genes involved in fatty acid transport, binding and activation, and β-oxidation (Bougarne et al. 2018).

The PPARα agonists that produce a response by binding to PPARα can be divided into exogenous and endogenous ligands. Exogenous ligands, also called peroxisome proliferators, are synthetic compounds including hypolipidemic agents such as clofibrate, fenofibrate, ciprofibrate, and WY-14643 (Pirinixic acid) (Mukherjee et al. 2008). Pemafibrate is a novel and highly selective PPARα modulator that strongly binds to PPARα and regulates the expression of target genes that are mainly related to lipid and glucose metabolism and decreases plasma triglyceride levels (Fruchart 2013; Blair 2017). Pemafibrate has been used as a therapeutic drug for patients with hyperlipidemia and/or metabolic associated fatty liver disease (Nakajima et al. 2021). A protective role of PPARα was observed using PPARα-null mice in experimentally induced ALD (Nakajima et al. 2004). Furthermore, it was reported that treatment with PPARα agonist WY-14643 induces peroxisomal fatty acid oxidation and inhibits ethanol-induced hepatic steatosis in mice (Xu et al. 2022). These data suggest that treatment with pemafibrate could also prevent hepatic steatosis and ALD during chronic ingestion of ethanol. In the present study, at the first phase, we fed rats with Lieber–DeCarli liquid diet containing ethanol for 4 weeks to produce hepatic steatosis (Lieber and DeCarli 1970). In the second phase, the rats fed with liquid diet containing ethanol have been treated with pemafibrate for another 4 weeks and evaluated whether pemafibrate could reduce hepatic steatosis and ALD.

## Materials and methods

### Animals

About 6 weeks old Wistar male rats (body weight: 160–180 g) were procured from Japan SLC, Inc. (Hamamatsu, Shizuoka, Japan). The animals were housed in temperature and humidity-controlled rooms in stainless steel wire mesh cages with 12 h light/dark cycles. They were allowed to have food and water ad libitum for one week before the start of experiments. The animal experiments were carried out with the Guide for the Care and Use of Laboratory Animals published by the US National Institutes of Health (NIH Publication No. 86-23, revised 1996). The protocol (#2022-29) was approved by the Animal Care and Research Committee of Kanazawa Medical University on the Ethics of Animal Experiments.

### Experimental design

All the animal experiments were carried out in the animal house and the adjacent laboratories at Kanazawa Medical University, Uchinada, Japan. A total of 30 rats were used in the study. About 7 weeks old albino male rats of Wistar strain (body weight: 225.3 ± 6.7 g) were divided into two groups. In order to induce hepatic steatosis, one group of 15 rats (ethanol group) was administered ethanol-containing liquid diet (36% of total calories), and another group of 15 rats (control group) was pair-fed with the control diet, where ethanol was replaced isocalorically with carbohydrate for a period of 4 weeks (Lieber and DeCarli 1970). Subsequently, either 0.5% aqueous methylcellulose solution (MC) or MC containing 0.3 mg pemafibrate/kg body weight were administered orally twice a day (around 10 AM and 6 PM) along with the liquid diet, employing a flexible tube (Cat No. KN-349-RB, Natsume, Bunkyo-ku, Tokyo, Japan) for another 4 weeks. Five rats each in the ethanol and control groups were sacrificed at the end of 4 weeks to demonstrate that steatosis has already been developed in the ethanol treated group. The remaining 20 rats were sacrificed at the end of 8 weeks to evaluate the effects of pemafibrate treatment. All the animals were anesthetized with isoflurane before euthanasia, and blood was collected from the right jugular vein. The animals were euthanized under deep anesthesia using a rodent guillotine, and the excess blood was allowed to drain out. The livers were excised quickly, weighed, and about 100 mg of liver tissue from the median lobe was cut into small pieces and fixed in RNA-later solution (Life Technologies, Tokyo, Japan) and stored at − 20 °C for PCR analysis. The median lobe of the liver tissue was cut into 3 mm pieces and instantly fixed in 10% phosphate-buffered formalin for histopathological and immunohistochemical studies. The remaining portion of the liver tissue was flash frozen in liquid nitrogen and stored at − 80 °C for biochemical analysis. Animal body weight was measured during the entire course of the study, and the liver wet weight to body weight ratio was calculated.

### Measurement of aspartate aminotransferase, alanine aminotransferase, total cholesterol and triglycerides in serum

Blood was allowed to clot at 37 °C for 3–5 h, and serum was separated after centrifugation at 3000 rpm in a clinical centrifuge. Aspartate aminotransferase (AST), alanine aminotransferase (ALT), total cholesterol, and triglycerides present in the serum were measured using an auto-analyzer for animal samples. Serum AST and ALT values are presented as International Units per liter (IU/L). Total cholesterol and triglycerides are presented as mg/100 mL of serum.

### Measurement of NAD and NADH concentrations in the hepatic tissue

Nicotinamide adenine dinucleotide (NAD) and its reduced form (NADH) are key cofactors in ethanol metabolism. Hepatic NAD and NADH concentrations in the liver tissue were measured based on a fluorometric technique employing NAD/NADH assay kit (EFND-100, BioAssay Systems, Hayward, CA, USA). About 20 mg of frozen liver tissue was homogenized with either 100 μL NAD extraction buffer for NAD determination or 100 μL NADH extraction buffer for NADH determination. NAD or NADH was extracted from the homogenized sample by heating at 60 °C for 5 min and then added 20 μL of assay buffer and 100 μL of neutralizing buffer. The samples were vortexed gently and centrifuged at 14,000×*g* for 5 min at 4 °C. Hepatic NAD and NADH were determined in the supernatant and presented as nmoles/100 mg liver wet tissue.

### Histopathological evaluation of the liver tissue

The formalin-fixed liver tissues were processed in an automatic tissue processor optimized for liver tissue. Then it was embedded in paraffin blocks and cut into sections of 5 μm thickness. The sections were stained with Hematoxylin and Eosin (H&E) and examined using an Olympus BX53 optical microscope attached with a DP 71 digital camera (Olympus Corporation, Tokyo, Japan) and captured the images.

The histopathological alterations during ethanol administration and treatment with methylcellulose or pemafibrate was scored as per the method of Bedossa et al. (Bedossa et al. 2012) and are reported as SAF score [steatosis, activity (comprises hepatocyte ballooning and lobular inflammation), and fibrosis]. The steatosis score (S) was assessed as the quantities of large or medium-sized lipid droplets, from 0 to 3 (S0: < 5%; S1: 5%–33%, mild; S2: 34%–66%, moderate; S3: > 67%, marked). Activity grade (A) was scored from 0 to 3 as A0 (no activity), A1 (mild activity), A2 (moderate activity), and A3 (severe activity). The stages of fibrosis (F) was assessed as follows; F0 (no fibrosis), F1 (portal and periportal fibrosis), F2 (bridging fibrosis), and F3 (advanced fibrosis and early cirrhosis). The average grade was calculated after examining 10 lobules on each liver section.

### Measurement of hepatic triglycerides

Triglycerides content in the rat liver tissue after the administration of ethanol and treatment with pemafibrate were quantified employing a colorimetric assay kit (Cat #10010303, Cayman Chemical, Ann Arbor, MI, USA) as per the manufacturer's protocol. In brief, about 100 mg frozen liver tissue in an eppendorf tube was homogenized in 500 µL of NP40 assay reagent (present in the kit) and centrifuged at 10,000×*g* at 4 °C for 10 min. The supernatant was collected in a new eppendorf tube. Different concentrations of triglyceride standard solutions and supernatant samples (10 µL each) were mixed with 150 µL of reconstituted enzyme assay mixture (supplied in the kit) and allowed to stand at room temperature for 60 min. The resultant color was read at 540 nm in a spectrophotometer. Hepatic triglyceride content was calculated based on the standard values and presented as mg/g of liver wet tissue.

### Measurement of mRNA levels of hepatic lipid metabolism related genes

Real-Time quantitative Reverse Transcription-polymerase chain reaction (Real-Time qRT-PCR) was performed to evaluate the rate of expression of hepatic lipid metabolism related genes such as PPARα, sterol regulatory element-binding protein-1 (SREBP-1), carnitine palmitoyltransferase 1a (CPT1A), CPT2, very-long chain acyl-CoA dehydrogenase (VLCAD), and acyl-CoA oxidase 1 (ACOX1) in the rat hepatic tissue. Total cellular RNA was isolated from the rat liver tissue using RNeasy Mini Kit (Qiagen, Valencia, CA, USA) as per the manufacturer’s protocol. The purity of the isolated RNA was evaluated using ultraviolet spectrometry and found that the A260: A280 ratio was > 1.8. About 0.5 μg of pure isolated RNA was reversely transcribed into complementary DNA (cDNA) using PrimeScript RT Master Mix (# RR036A, TaKaRa, Shiga, Japan) in a total volume of 10 μL with RNAse free H_2_O at 37 °C for 15 min. The primer set for each hepatic lipid metabolism related genes (TaqMan) was procured from Applied Biosystems (Carlsbad, CA, USA). The expression rate of each hepatic lipid metabolism related genes was quantified by using TaqMan real-time qRT-PCR (QuantStudio 3 Real-Time PCR system, Applied Biosystems). Each reaction was multiplexed with β-actin (#NM_031144.3) as a housekeeping gene, and all the data were normalized based on the expression levels of β-actin. All the samples were run in triplicate. The quantitative PCR was performed as follows: the samples were initially denatured at 95 °C for 20 s (1 cycle), then denatured at 95 °C for 15 s and annealed at 60 °C for 20 s (40 cycles), a final melting curve at 50 °C for 1 min (1 cycle), and cooling to 25 °C (1 cycle).

### Western blotting of molecules involved in lipid metabolism

We carried out Western blotting to assess the protein levels of molecules involved in lipid metabolism such as PPARα, CPT1A, CPT2, VLCAD, and ACOX1 in the rat liver after ethanol administration and treatment with pemafibrate. About 100 mg of liver tissue was homogenized in 1 ml of cold 50 mM Tris–HCl buffer (pH 8.0) containing 150 mM NaCl, 1 mM EDTA, and 1% Triton X-100 along with protease inhibitors. The homogenates were centrifuged at 10,000×*g* for 10 min at 4 °C and the supernatant was collected. Protein concentrations in the supernatants were determined using a protein assay kit. The proteins were denatured and resolved on 4–15% SDS–polyacrylamide gradient gel and transblotted to activated polyvinylidene difluoride membranes (Bio-Rad, Hercules, CA, USA). The non-specific binding sites were blocked with a blocking agent (Nacalai Tesque, Kyoto, Japan), and the membranes were incubated overnight on a rocker at 4 °C with specific antibodies. The antibodies against PPARα and β-actin were procured from Santa Cruz Biotechnology (Dallas, Texas, USA). The antibodies to CPT1A, CPT2, VLCAD, and ACOX1 were obtained from Proteintech (Rosemont, IL, USA). The membranes were washed thrice and incubated with respective HRP-conjugated secondary antibodies (Promega, Madison, WI, USA). The membranes were washed again and treated with Pierce chemiluminescent substrate (Thermo Fisher Scientific, Waltham, MA, USA), and the images were captured using ImageQuant LAS 4000 (GE Healthcare, Piscataway, NJ, USA). The membranes were re-probed using Western reprobe buffer (#786-119, Gbiosciences, St. Louis, MO, USA) for β-actin content to demonstrate that samples loaded in all the lanes contain equal amounts of protein. The Western blot images were quantified using Gel-Pro analyzer software (Media Cybernetics, Silver Spring, MD).

### Data analysis and statistics

The arithmetic mean and standard deviation (SD) were calculated for all the data and presented as Mean ± SD. The data were analyzed and compared using Student's t*-*test at the 4th week (control and ethanol groups) and one-way analysis of variance (ANOVA) at the 8th week (more than two groups of data with one independent variable). Bonferroni post-hoc test was used to determine the level of significance between each group. A value of P < 0.05 was considered statistically significant.

## Results

### Alteration of animal body weight, liver weight and liver weight to body weight ratio

**Fig. 1:**
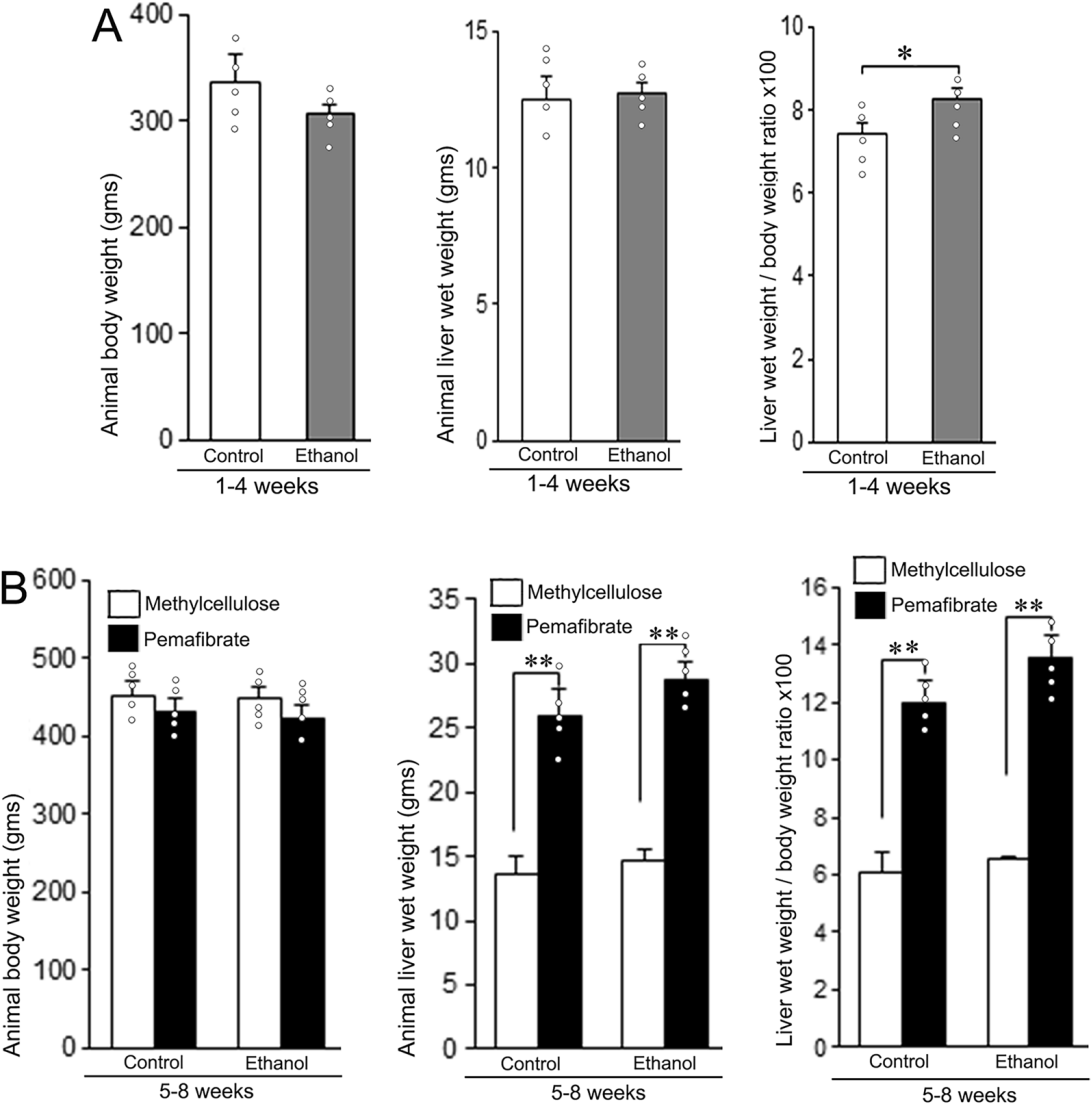
Body weight, liver wet weight, and liver wet weight to body weight ratio of animals fed with control diet or ethanol diet with methylcellulose or pemafibrate. **A** Animals fed with control or ethanol diet for 4 weeks. There was no alteration in either body weight or liver weight. The liver weight to body weight ratio was significantly higher compared to the control group. **B** Animals treated with methylcellulose or pemafibrate for 4 weeks. The liver weight and liver weight to body weight ratio of both control and ethanol groups treated with pemafibrate increased significantly compared to the animals administered with methycellulose. Data are mean ± SD (N = 5). *P < 0.05 and **P < 0.01

As depicted in Fig. 1A, there was no difference in animal body weight or liver weight between the control and ethanol groups at the 4th week. Furthermore, the animal body weight has not been changed after the treatment with methylcellulose or pemafibrate at the 8th week. However, the liver weight has been significantly increased in pemafibrate group compared to methylcellulose group in both control and ethanol diet treated animals (Fig. 1B). Similarly, the liver weight to body weight ratio has been markedly increased in pemafibrate treated animals in both control and ethanol diet groups at the 8th week (Fig. 1B).

### Effect of pemafibrate on serum levels of ALT, AST, triglycerides and total cholesterol

**Fig. 2:**
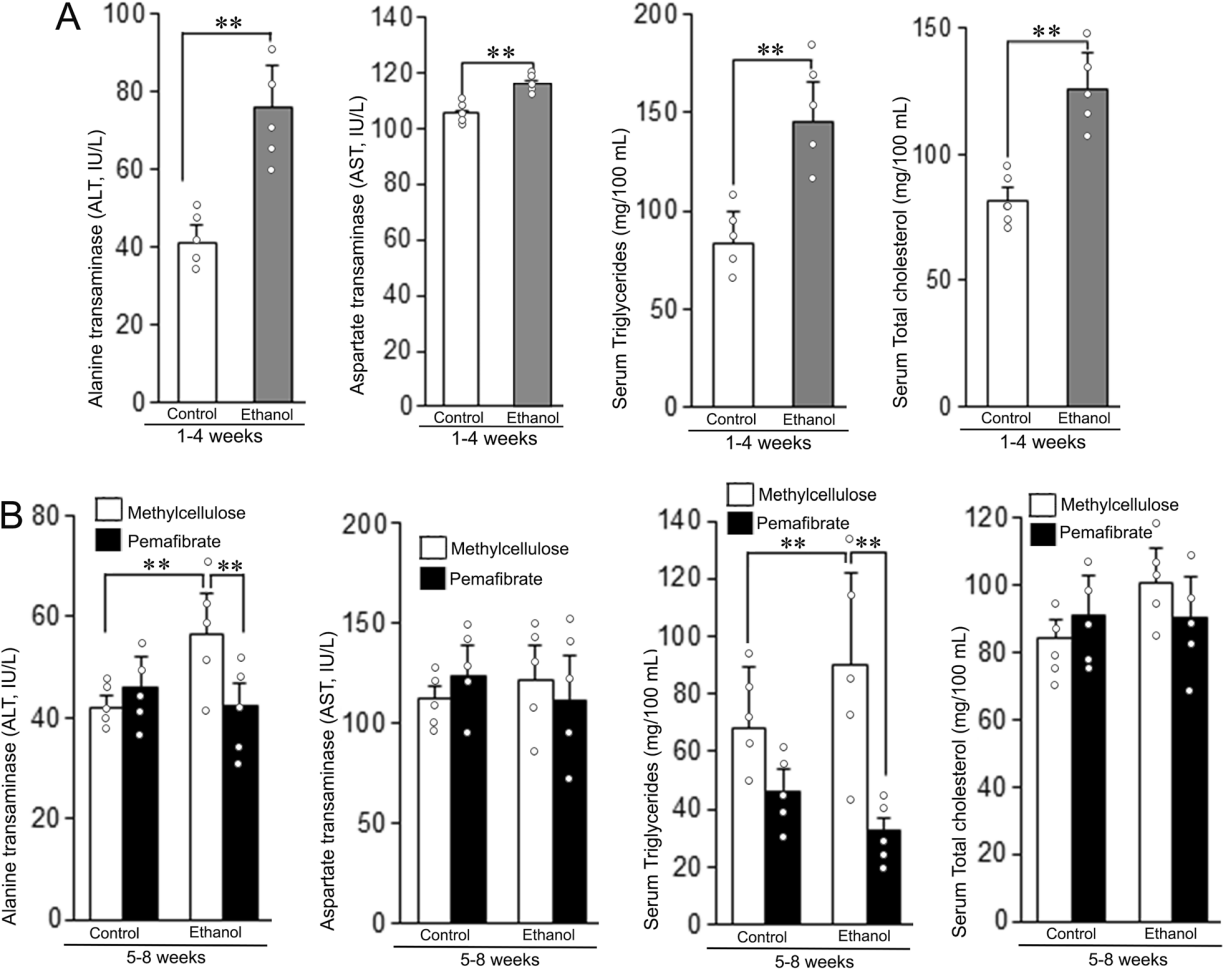
Serum levels of alanine aminotransferase (ALT), aspartate aminotransferase (AST), triglycerides (TG), and total cholesterol in the animals fed with control diet or ethanol diet with methylcellulose or pemafibrate. **A** Animals fed with control or ethanol diet for 4 weeks. Serum levels of ALT, AST, TG, and cholesterol were significantly increased in the ethanol group compared to the control group. **B** Animals treated with methylcellulose or pemafibrate for 4 weeks. Serum levels of ALT and TG were significantly increased in the ethanol group of animals administered with methylcellulose compared to the similarly treated controls, which were markedly reduced after the treatment with pemafibrate. Data are mean ± SD (N = 5). **P < 0.01

The serum levels of ALT, AST, triglycerides, and total cholesterol after the administration of ethanol and treatment with pemafibrate are presented in Fig. 2. There was significant increase (P < 0.01) in the levels of ALT, AST, triglycerides and total cholesterol in the ethanol group compared to the respective control group at the 4th week. Treatment with pemafibrate resulted in significant decrease (P < 0.01) of ALT and triglycerides level in the ethanol group compared to the animals treated with methylcellulose (Fig. 2B).

### Treatment with pemafibrate increased hepatic NAD and NADH levels

**Fig. 3:**
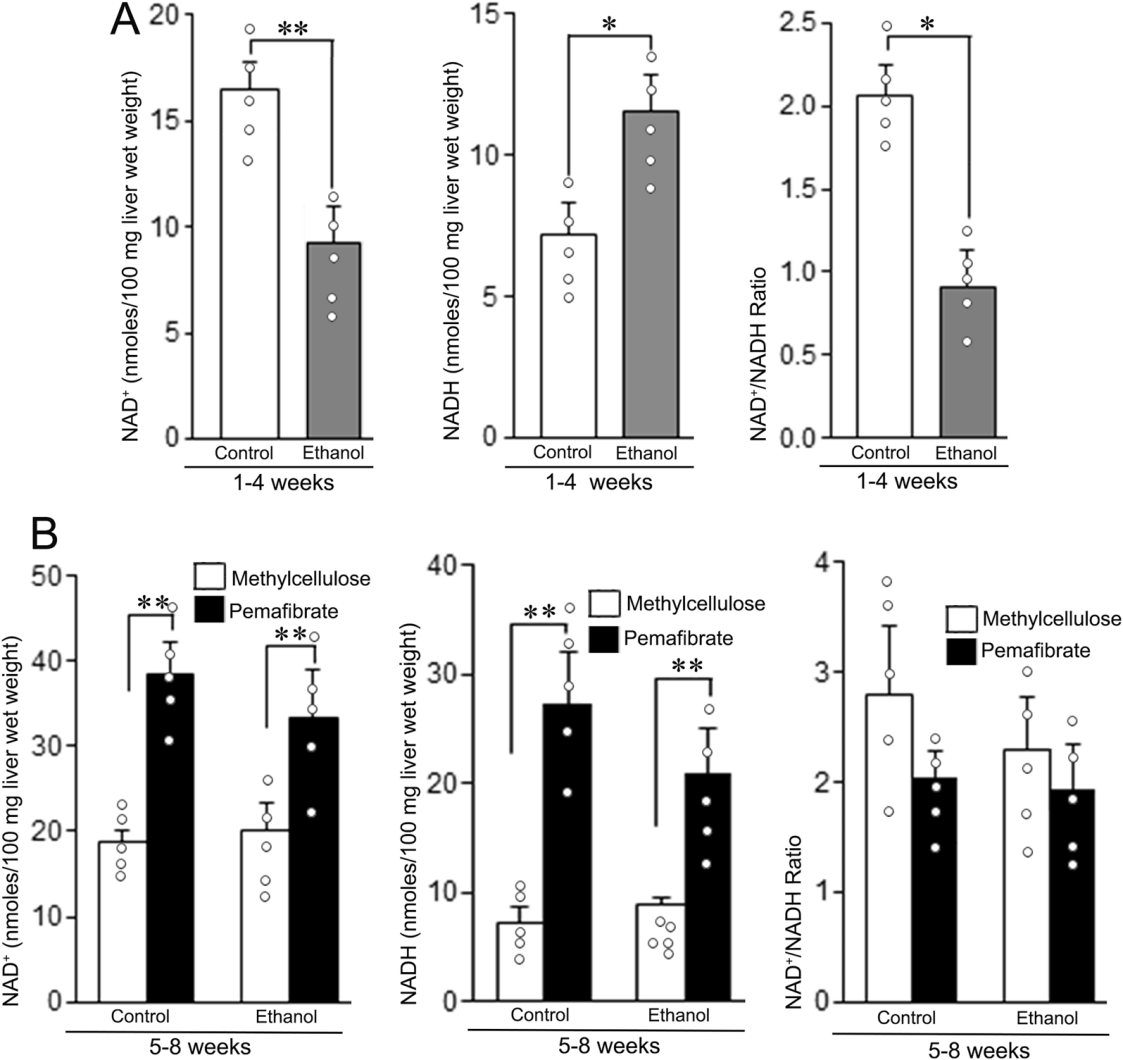
Levels of NAD, NADH, and NAD/NADH ratio in the livers of rats fed with control diet or ethanol diet with methylcellulose or pemafibrate. **A** Animals fed with control or ethanol diet for 4 weeks. In the ethanol group, hepatic NAD content decreased and NADH content increased compared with the respective control group, resulting in a significant decrease of NAD^+^/NADH ratio. **B** Animals treated with methylcellulose or pemafibrate for 4 weeks. Hepatic NAD^+^ and NADH content in both the control and ethanol groups treated with pemafibrate increased significantly compared to the respective methylcellulose group. Hepatic NAD^+^/NADH ratio in both the control and ethanol groups significantly reduced compared to the respective methylcellulose group. Data are mean ± SD (N = 5). *P < 0.05 and **P < 0.01

Hepatic NAD and NADH content during the administration of ethanol and after the treatment with pemafibrate are presented in Fig. 3. At the 4th week of ethanol feeding, the NAD content in the liver tissue was decreased due to the extensive consumption of NAD by dehydrogenases for the metabolic degradation of ethanol. The NADH content in turn increases (Fig. 3A). Treatment with pemafibrate resulted in significant increase of NAD and NADH levels in both the control and ethanol groups (Fig. 3B). This resulted in the restoration of normal NAD^+^/NADH ratio in pemafibrate treated rats in both the control and ethanol groups.

### Histopathological alterations of liver tissue during ethanol feeding and after the treatment with pemafibrate.

**Fig. 4:**
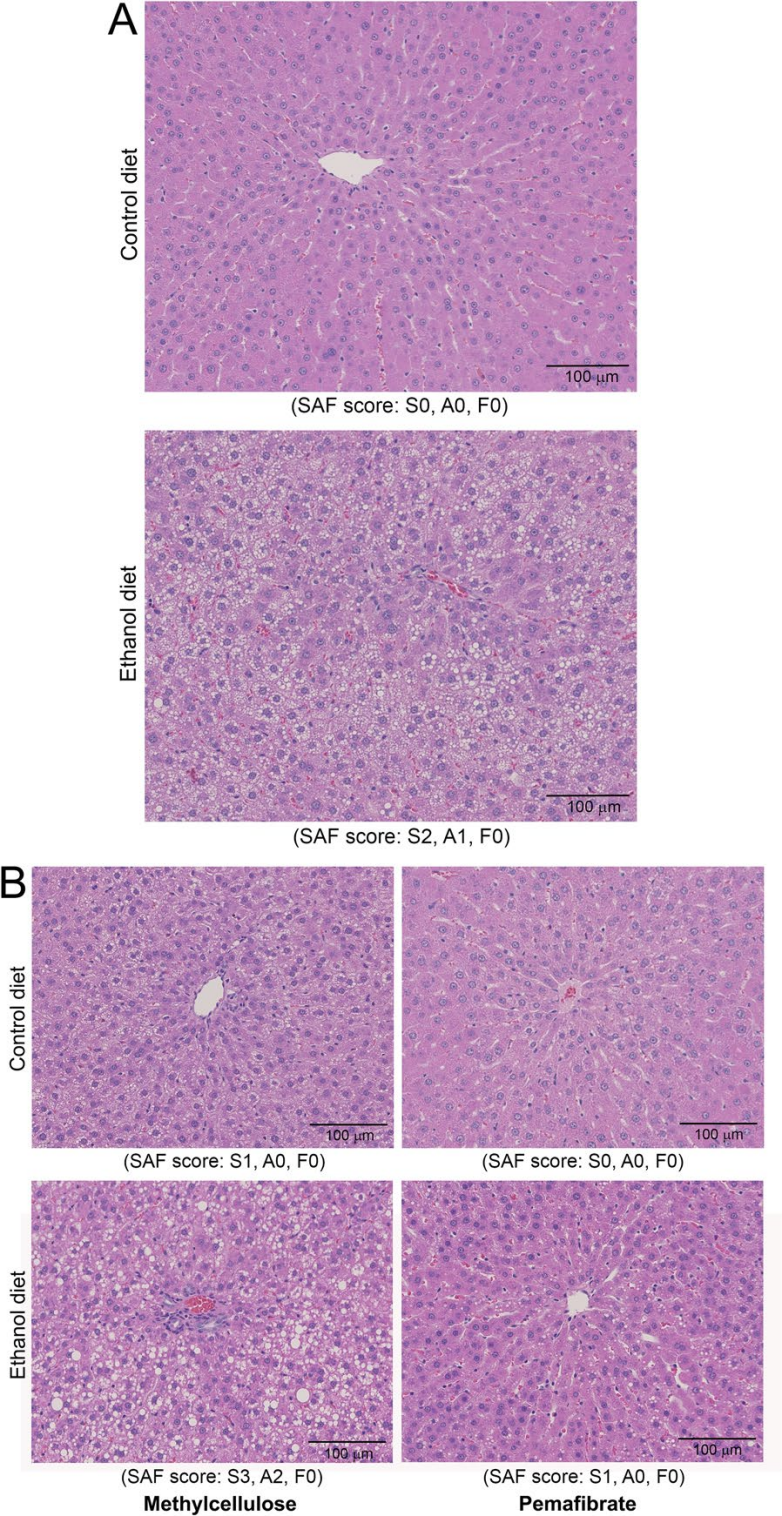
Hematoxylin and Eosin staining of rat livers fed with control diet or ethanol diet and after treatment with methylcellulose or pemafibrate. **A** Animals fed with control diet or ethanol diet for 4 weeks. The rats fed with the control diet did not show any histopathological alterations. Significant fatty degeneration, mild hepatic inflammation, and ballooning of hepatocytes were present in rat livers fed with ethanol diet. **B** Animals treated with methylcellulose or pemafibrate for 4 weeks. Mild fatty degeneration was observed in the control group of animals administered with methylcellulose, which were absent in pemafibrate treated group. Extensive fatty degeneration with deposition of large fat globules, moderate hepatic inflammation, and ballooning of hepatocytes were present in the ethanol group. Treatment with pemafibrate prevented significant deposition of fat globules in the hepatic tissue, hepatic inflammation, and ballooning of hepatocytes. However, small and intermittent fat globules were present. All histopathological images are original magnification, ×100

Histopathological changes in the liver tissue during ethanol administration and after the treatment with pemafibrate as well as methylcellulose are presented in Fig. 4. At the 4th week of ethanol feeding with Lieber-DeCarli liquid diet, extensive fatty degeneration, mild hepatic inflammation, and hepatocyte ballooning were present, especially in the centrilobular areas (Fig. 4A). There was enlargement of nuclei and mitosis, indicating hepatocyte regeneration. At the 8th week of ethanol feeding, there was extreme fatty degeneration with deposition of large fat globules in the hepatocytes with moderate hepatic inflammation (Fig. 4B). Treatment with pemafibrate prevented more than 90% of fatty degeneration in the hepatocytes. Only small fat droplets were present. Hepatic inflammation was completely absent. A slight fatty degeneration was present in the animals treated with the vehicle methylcellualose, which completely disappeared in pemafibrate treated animals (Fig. 4B). The SAF score is presented as 0–3 grades below the representative image from each group in Fig. 4.

### Effect of pemafibrate on hepatic triglyceride levels

**Fig. 5:**
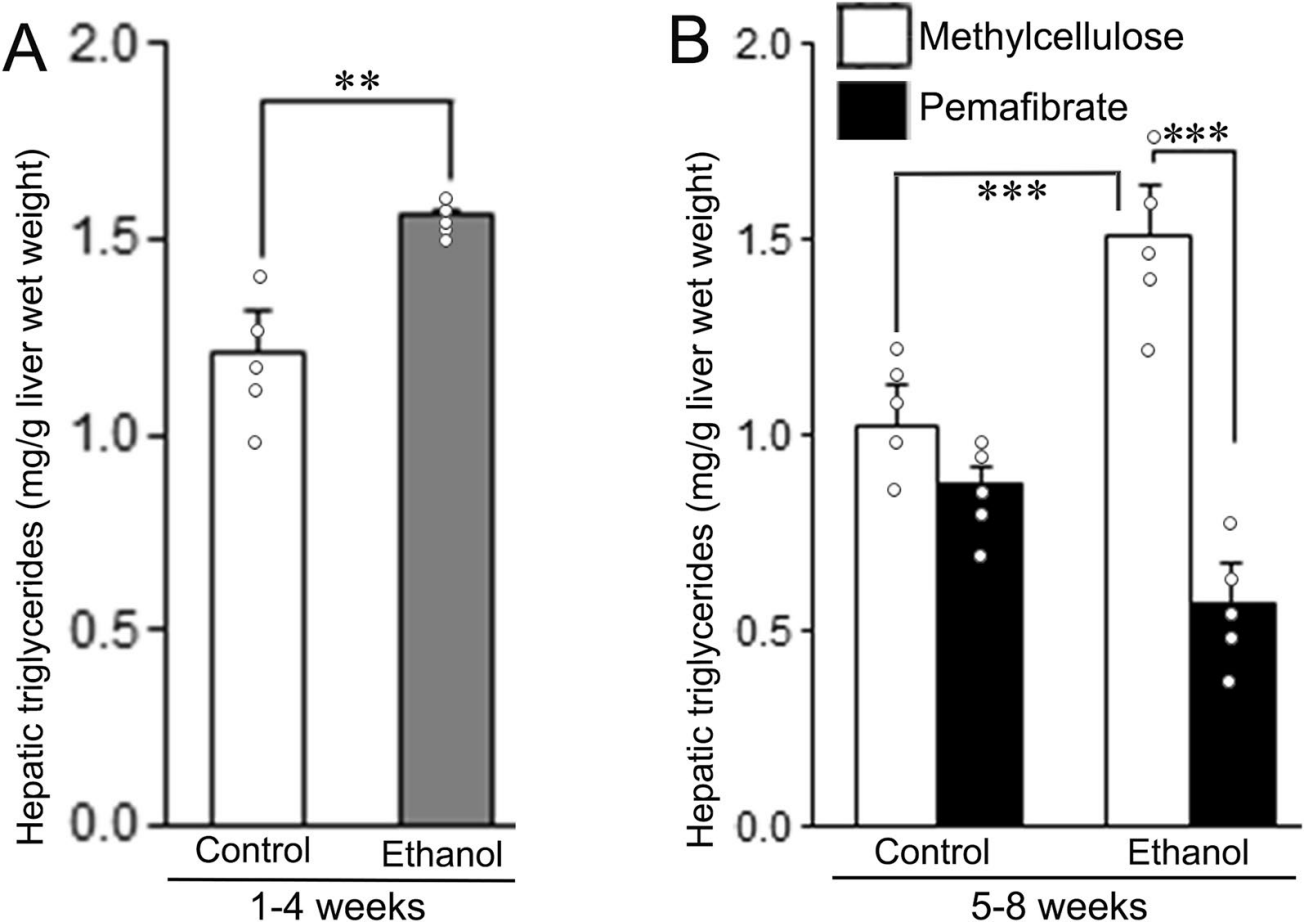
Hepatic triglycerides content in the livers of rats fed with control diet or ethanol diet and after treatment with methylcellulose or pemafibrate. **A** Animals fed with control diet or ethanol diet for 4 weeks. There was an increase of hepatic triglyceride content in the ethanol group. **B** Animals treated with methylcellulose or pemafibrate for 4 weeks. Hepatic triglyceride levels were significantly decreased in pemafibrate treated control and ethanol groups compared to the respective methylcellulose group. Data are mean ± SD (N = 5). *P < 0.05 and ***P < 0.001

The effect of pemafibrate on hepatic triglyceride levels during ethanol feeding to rats is presented in Fig. 5. Ethanol feeding lead to an increase in hepatic triglyceride levels at the 4th week (Fig. 5A) and at the 8th week (Fig. 5B). Treatment with pemafibrate resulted in a marked decrease of hepatic triglyceride levels compared to the methylcellulose group in ethanol fed animals (Fig. 5B).

### Effect of pemafibrate on expression of hepatic lipid metabolism related genes

**Fig. 6:**
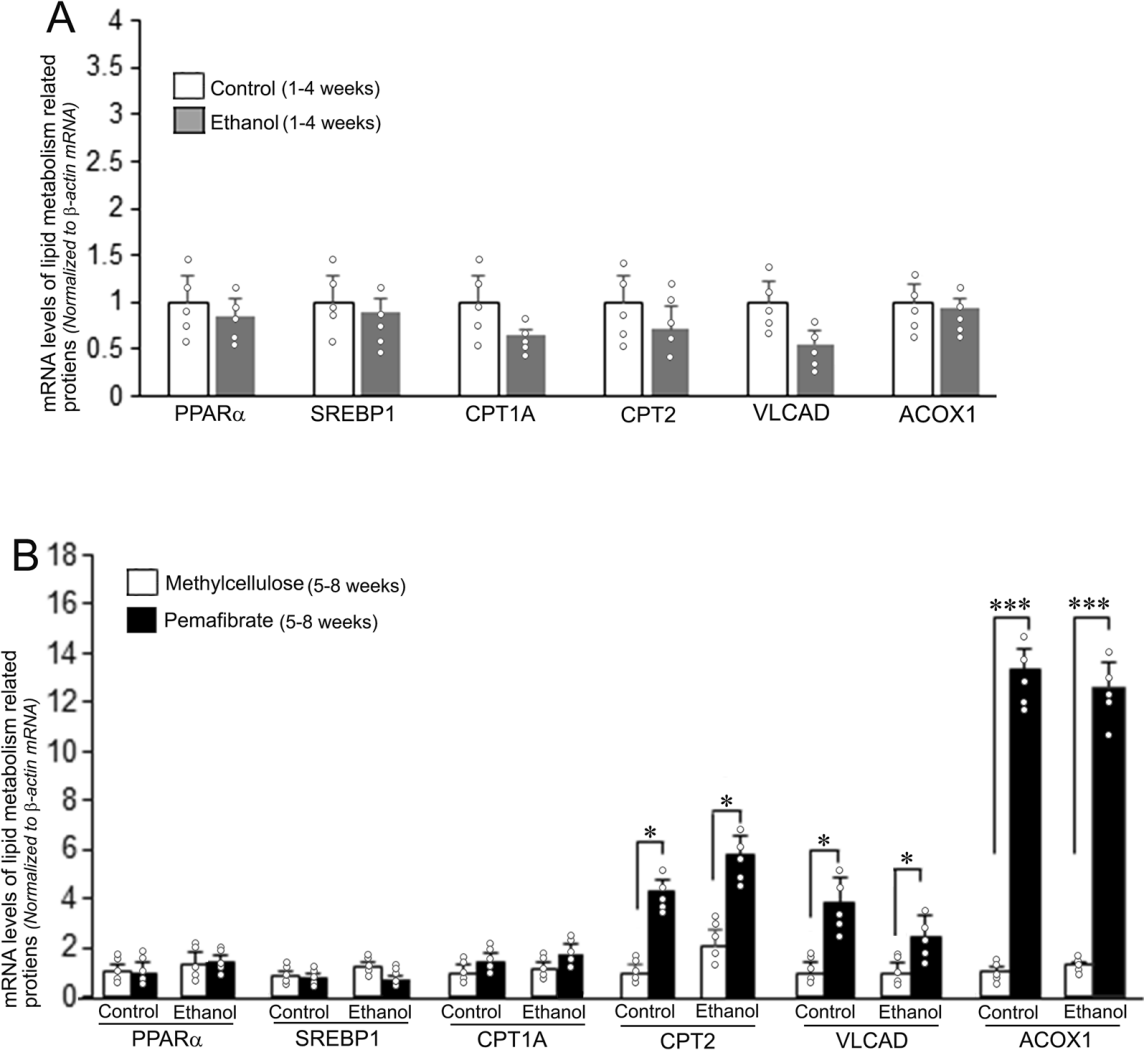
mRNA levels of lipid metabolism related molecules in the livers of rats fed with control diet or ethanol diet with methylcellulose or pemafibrate. **A** Animals fed with control or ethanol diet for 4 weeks. There was no significant difference in the expression rate of hepatic lipid metabolism related genes between the control and ethanol treated groups. **B** Animals treated with methylcellulose or pemafibrate for 4 weeks. The mRNA levels of CPT2, VLCAD, and ACOX1 were significantly increased in animals treated with pemafibrate in both the control and ethanol groups compared to the rats treated with methylcellulose in the respective groups. Data are mean ± SD (N = 5). *P < 0.05 and ***P < 0.001

The effect of pemafibrate on the rate of expression of major hepatic lipid metabolism related genes evaluated employing qRT-PCR is presented in Fig. 6. There was no significant alteration in any of the genes evaluated at the 4th week of ethanol administration (Fig. 6A). Treatment with pemafibrate resulted in significant increase in the mRNA levels of CPT2, VLCAD, and ACOX1 in both the control and ethanol groups compared to the methylcellulose treated animals in the respective groups (Fig. 6B). The maximum increase (over 12 fold) was observed in the case of ACOX1 gene.

### Effect of pemafibrate on molecules involved in lipid metabolism

**Fig. 7:**
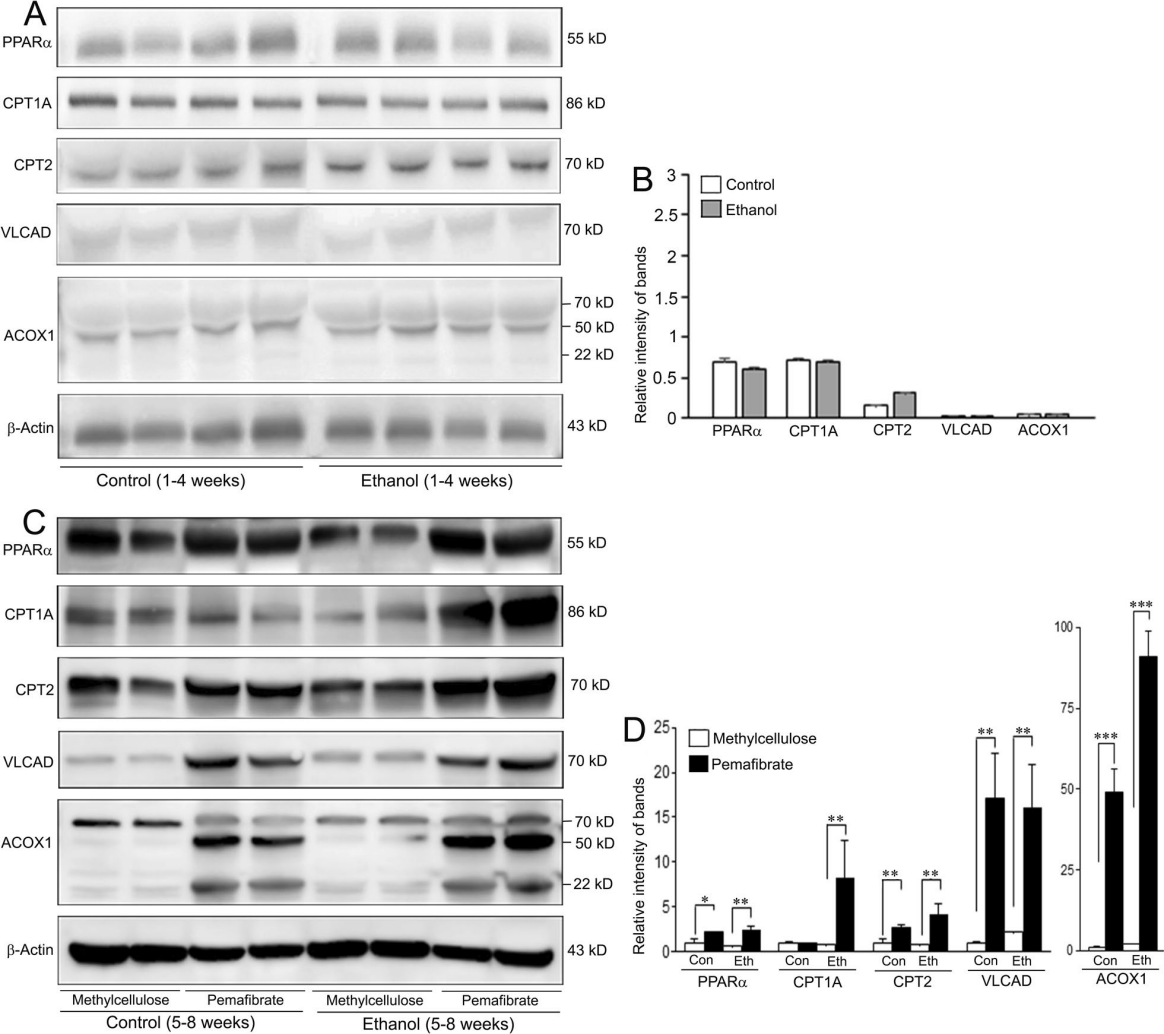
Levels of lipid metabolism related proteins in the livers of rats fed with control diet or ethanol diet with methylcellulose or pemafibrate. **A**, **B** Animals fed with control or ethanol diet for 4 weeks. **A** Western blots for proteins involved in lipid metabolism. Five samples were run for each molecule. **B** Quantification of Western blot images. There was no difference in the rate of expression of proteins involved in hepatic lipid metabolism between the control and ethanol groups. Data are mean ± SD of 5 independent samples. **C**, **D**) Animals treated with methylcellulose or pemafibrate for 4 weeks. **C** Western blot images of protein molecules related to lipid metabolism. **D** Quantification of Western blot images. Protein levels of PPARα, CPT1A, CPT2, VLCAD, and ACOX1 were significantly increased in pemafibrate treated rats of both the control and ethanol groups compared to the animals administered with methylcellulose in the respective groups. The Western blot images were quantified using the Gel-Pro analyzer software. Data are mean ± SD (N = 5). *P < 0.05, **P < 0.01, and ***P < 0.001

The effect of pemafibrate on major molecules involved in lipid metabolism evaluated employing Western blotting is presented in Fig. 7. Quantification of Western blot images did not show significant difference between the control and ethanol groups in any of the protein molecules studied at the 4th week (Fig. 7B). However, there was marked and significant increase in protein levels of PPARα, CPT1A, CPT2, VLCAD, and ACOX1 after pemafibrate treatment in both the control (except in the case of CPTA1) and ethanol groups at the 8th week, compared to the animals treated with methylcellulose in the respective groups (Fig. 7D).

## Discussion

Alcohol-associated liver disease is the most prevalent type of chronic liver disease that begins with simple steatosis and progresses to steatohepatitis, fibrosis, and cirrhosis (Jeon and Carr 2020). Ethanol is mainly metabolized by alcohol dehydrogenase (ADH), which requires NAD^+^ to accept the released H^+^ from ethanol and to form NADH. Thus, ethanol metabolism by ADH utilizes NAD^+^, which leads to increased ratio of NADH/NAD^+^ in the liver. Fatty acid oxidation (FAO) involves several steps of dehydrogenation, which also require NAD^+^ to accept the released H^+^ from fatty acids. Therefore, an increased ratio of NADH/NAD^+^ inhibits FAO and leads to the deposition of fat in the hepatic tissue and is called fatty liver. PPARα is a ligand binding activated transcription factor that mainly regulates lipid metabolism in the liver (Lin et al. 2022). Activation of PPARα promotes uptake, utilization, and catabolism of fatty acids through upregulation of several genes involved in fatty acid mobilization, binding, and activation, as well as peroxisomal and mitochondrial fatty acid β-oxidation (Pawlak et al. 2015). Pemafibrate is a highly selective PPARα modulator, and treatment with pemafibrate reduces serum triglycerides and increases high density lipoproteins (HDL) cholesterol in patients with dyslipidemia and metabolic diseases without deleterious side effects (Yamashita et al. 2020). In addition, pemafibrate is metabolized in the liver and excreted into the bile rather than through the kidney, which makes it safe for patients with impaired renal function. In the present study, we evaluated whether treatment with pemafibrate could also prevent hepatic steatosis during chronic ingestion of ethanol, where the mechanism of the deposition of fat in the liver is different from metabolic liver diseases (Saito et al. 2024). This is the first study to demonstrate the effects of pemafibrate on alcoholic liver disease in a rat model.

In the current study, we used Lieber–DeCarli liquid diet containing ethanol to produce hepatic steatosis. Histopathological examination of the liver tissue confirmed well developed steatosis after 4 weeks of ethanol feeding. In addition, the effect of ethanol feeding was demonstrated by biochemical alterations such as increased levels of serum AST, ALT, triglycerides, and total cholesterol. Furthermore, the hepatic ethanol metabolism led to alteration of redox state, which results in increased NADH/NAD^+^ ratio (decreased NAD^+^/NADH ratio) in the liver. Real-Time qPCR and Western blotting showed no alteration in either mRNA or protein levels of the major genes involved in hepatic lipid metabolism such as PPARα, SREBP1, CPT1A, CPT2, VLCAD, and ACOX1, after 4 weeks of the ethanol feeding. These data indicate that the pathogenesis of hepatic steatosis as well as the increased hepatic triglyceride content could be due to the alteration of redox state, which decreases the activity of citric acid cycle as well as β-oxidation of fatty acids.

**Fig. 8:**
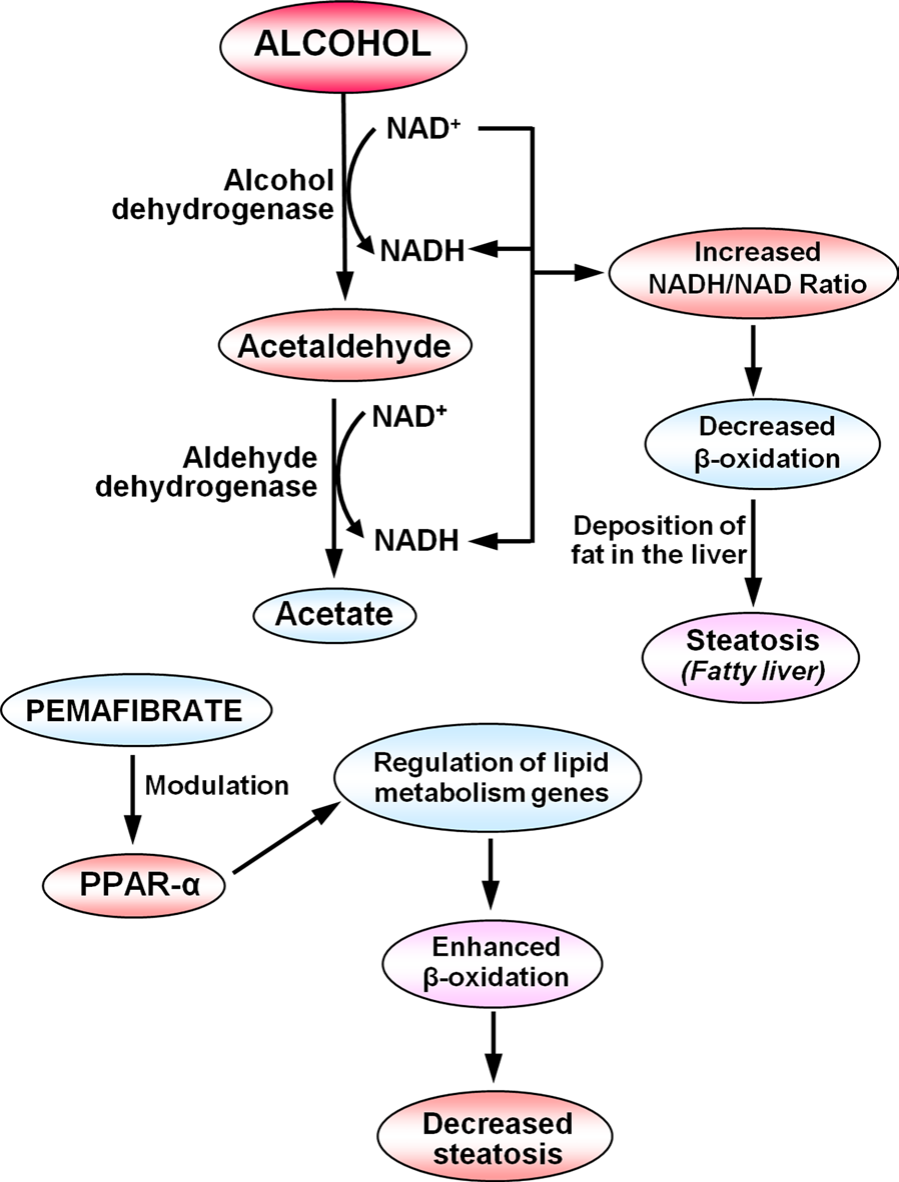
Schematic presentation of the metabolism of ethanol and the related increase of NADH/NAD ratio leading to decreased β-oxidation of fatty acids and subsequent deposition of fat in the liver (steatosis). Pemafibrate binds to PPAR-α and modulates the expression of genes involved in lipid metabolism that results in enhanced β-oxidation, thus decreasing plasma triglyceride levels and hepatic steatosis

The reported normal concentrations of NAD^+^ and NADH in rat liver ranged from 15.9 to 79.6 (n = 16) and 6.5 to 26.5 (n = 12) nmoles/100 mg liver tissue, respectively (Azouaoui et al. 2464). In the current study, we obtained a mean NAD^+^ level of 17.1 and a mean NADH level of 7.2 nmoles/100 mg liver tissue at the 4th week in control animals. Ethanol feeding resulted in a significant reduction of hepatic NAD^+^ levels, which in turn caused a marked elevation of NADH levels, leading to a decrease in NAD^+^/NADH ratio. As we discussed in our recent review, a reduced NAD^+^/NADH ratio or elevated NADH/NAD^+^ ratio consequently inhibits fatty acid oxidation, which results in the deposition of fat in the liver, leading to fatty liver or steatosis (Lu and George 2024). Therefore, regular drinking or consumption of alcohol (ethanol) can lead to simple steatosis, which progresses to alcohol-associated liver disease that could further aggravate and develop liver cirrhosis (Jeon and Carr 2020). Ethanol is metabolized by alcohol dehydrogenase, microsomal ethanol oxidation system (MEOS), mainly cytochrome P450 2E1 (CYP2E1), and catalase (Lu and George 2024). All these metabolic reactions produce acetaldehyde (CH_3_CHO), which is further metabolized into acetate by aldehyde dehydrogenase (ALDH), which also requires NAD^+^ to accept the released H^+^ from acetaldehyde and to form NADH. This further depletes NAD^+^ levels and increases NADH, leading to a decreased NAD^+^/NADH ratio. Figure 8 elucidates the steps involved in the metabolic degradation of ethanol into acetaldehyde and acetate, leading to increased NADH/NAD^+^ ratio as well as deposition of fat in the liver.

Pemafibrate (Mol weight 490.55), a PPARα agonist, is an approved hypolipidemic agent mainly used for the treatment of atherosclerotic cardiovascular diseases in Japan. A comparative study to evaluate the efficacy and safety of pemafibrate over fenofibrate to treat dyslipidemia indicated that pemafibrate is superior to fenofibrate to reduce serum triglyceride levels without affecting liver and renal functions (Ishibashi et al. 2018). Recently, it was reported that a PPARα agonist, WY-14643, could inhibit ethanol-induced fat deposition in mice liver (Xu et al. 2022). They used PPARα^+/+^ mice that are generated after crossbreeding PPARα^−/−^ mice with wild-type C57BL/6J mice and fed with Lieber-DeCarli liquid diet containing WY-14643. Since WY-14643 (Pirinixic acid) is a well established and highly selective PPARα agonist, treatment with WY-14643 could prevent hepatic steatosis during ethanol ingestion (Fischer et al. 2003; Chen et al. 2021). However, WY-14643 also serves as agonists and antagonists for several molecules and pathways and may not be suitable to use as a drug in humans (Sawaya et al. 2016; Yang et al. 2022). In the current study, before starting the pemafibrate administration, we fed the rats with ethanol containing liquid diet for 4 weeks in order to produce hepatic steatosis and raise triglyceride levels. During pemafibrate administration, we continued the ethanol ingestion into the rats in order to maintain steatosis and increased triglyceride levels in the control animals. In the pemafibrate treated group, hepatic steatosis disappeared, hepatic triglyceride levels significantly reduced, and the serum AST, ALT, and triglycerides were returned to the normal values. Therefore, pemafibrate could be used as a potent therapeutic agent to prevent hepatic steatosis, elevation of triglycerides, and the related biochemical alterations during chronic ethanol consumption.

Oxidation of fatty acids is pivotal to provide energy for innumerable biochemical reactions and also to prevent fat deposition in the body. Regular alcohol consumption will lead to impairment of fatty acid oxidation, which results in decreased energy supply and deposition of fat in the liver. It is well established that PPARα is a transcription factor that promotes the expression of several genes involved in hepatic lipid metabolism (Janssen et al. 2015). Since PPARα regulates the expression of several genes that are mainly responsible for lipid and glucose metabolism in the liver, we measured mRNA and protein levels of the major molecules involved in hepatic lipid metabolism. There was no alteration either in the mRNA or protein levels of PPARα, SREBP1, CPT1A, CPT2, VLCAD, or ACOX1 after 4 weeks of ethanol administration, which indicates that ethanol has no effect on any of these molecules. On the other hand, at the 8th week, there was marked increase in the mRNA levels of CPT2, VLCAD, and ACOX1 in both the control and ethanol groups of pemafibrate treated animals. Western blotting demonstrated marked and significant increase in the protein levels of PPARα, CPT1A, CPT2, VLCAD, and ACOX1 after pemafibrate treatment in both the control (except in the case of CPTA1) and ethanol groups of pemafibrate fed animals. These data clearly indicate that pemafibrate upregulates the molecules involved in hepatic lipid metabolism, which drives fatty acid oxidation and prevents deposition of fat in the liver.

## Conclusions

The data of the present study demonstrated that pemafibrate modulates PPARα and upregulates molecules involved in hepatic lipid metabolism both at gene and protein levels. In addition, treatment with pemafibrate increased hepatic NAD and NADH levels and markedly reduced the NAD^+^/NDAH ratio, which was significantly elevated during ethanol feeding. Furthermore, pemafibrate administration reduced hepatic triglyceride levels and prevented the deposition of fat globules within the hepatocytes. Therefore, pemafibrate may be used as a potent therapeutic agent to prevent hepatic steatosis and related adverse events during chronic consumption of alcohol.

## Supplementary Information

Supplementary Material 1.

## Data Availability

The data will be available for verification upon request. All original Western blot images are uploaded as a supplementary PDF file.

## Abbreviations

ACOX1: Acyl-CoA oxidase 1
ADH: Alcohol dehydrogenase
ALD: Alcohol-associated liver disease
ALDH: Aldehyde dehydrogenase
ALT: Alanine aminotransferase
ASH: Alcohol-associated steatohepatitis
AST: Aspartate aminotransferase
CPT1A: Carnitine palmitoyltransferase 1a
CPT2: Carnitine palmitoyltransferase 2
CYP2E1: Cytochrome P450 2E1
EDTA: Ethylene diamine tetraacetic acid
FAO: Fatty acid oxidation
HDL: High density lipoproteins
H&E: Hematoxylin and Eosin
HRP: Horseradish peroxidase
MC: Methylcellulose
MEOS: Microsomal ethanol oxidation system
NAD: Nicotinamide adenine dinucleotide
NADH: Nicotinamide adenine dinucleotide hydrogen (reduced form)
PPARs: Peroxisome proliferator-activated receptors
PPARα: Peroxisome proliferator-activated receptor alpha
PVDF: Polyvinylidene difluoride
qRT-PCR: Quantitative reverse transcription polymerase chain reaction
SDS: Sodium dodecyl sulfate
SDS-PAGE: Sodium dodecyl sulfate polyacrylamide gel electrophoresis
SREBP-1: Sterol regulatory element-binding protein-1
VLCAD: Very-long chain acyl-CoA dehydrogenase
