# Supplementary material for: Pemafibrate modulates peroxisome proliferator-activated receptor alpha and prevents alcohol-associated liver disease in rats
Source: Mol Med. 2025 Apr 22;31:145. doi: 10.1186/s10020-025-01210-9 (PMC12012945; doi:10.1186/s10020-025-01210-9)

Western Blots (1-4 weeks)

PPARα

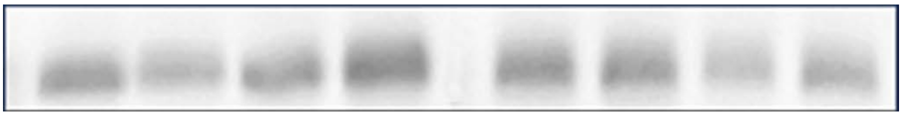

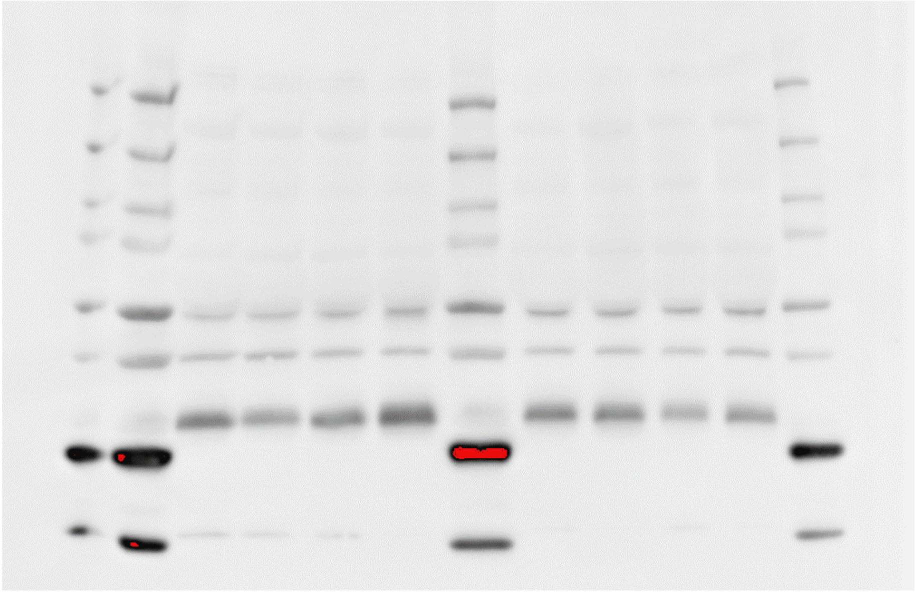

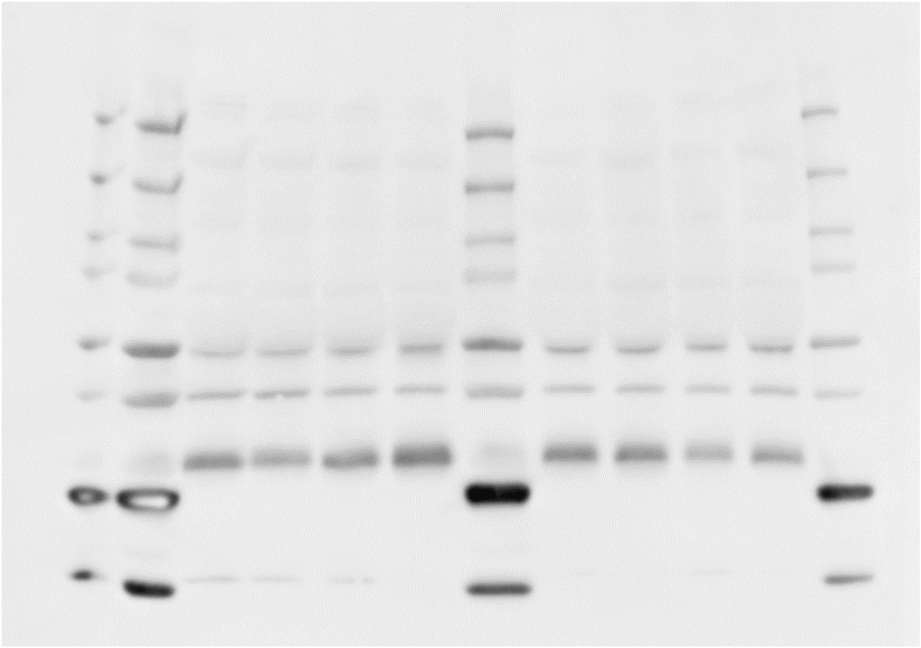

Western Blots (1-4 weeks)

CPT1A

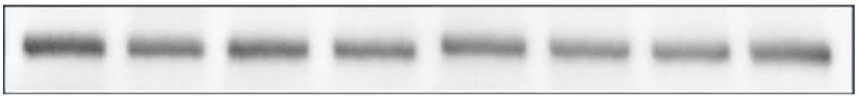

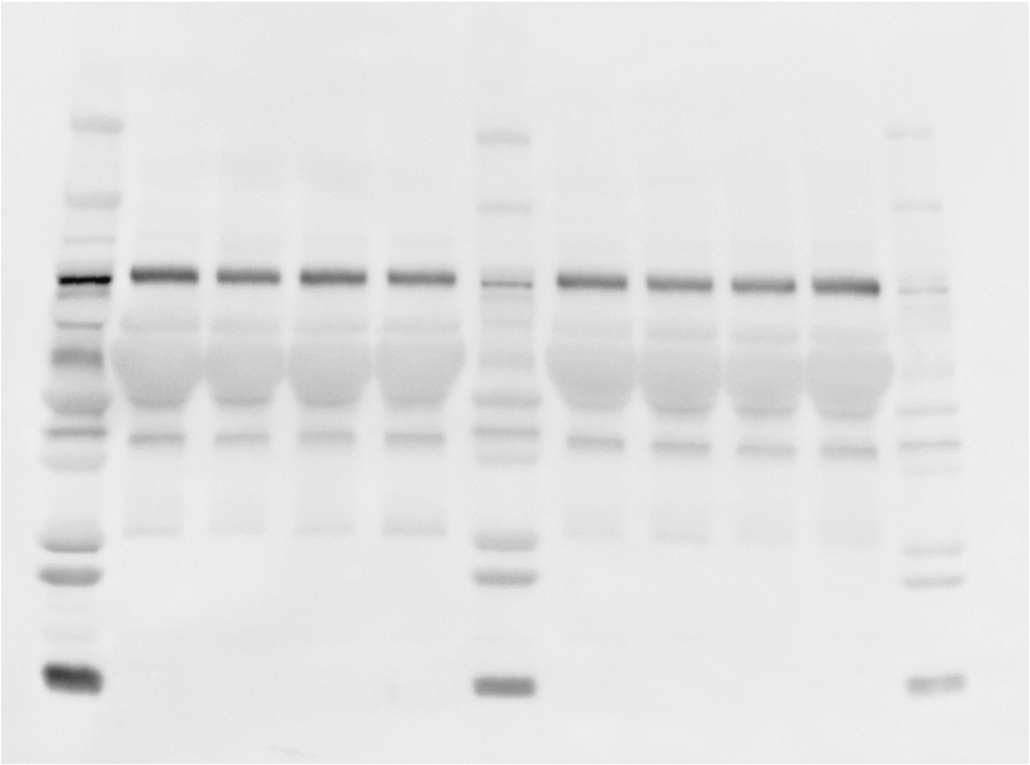

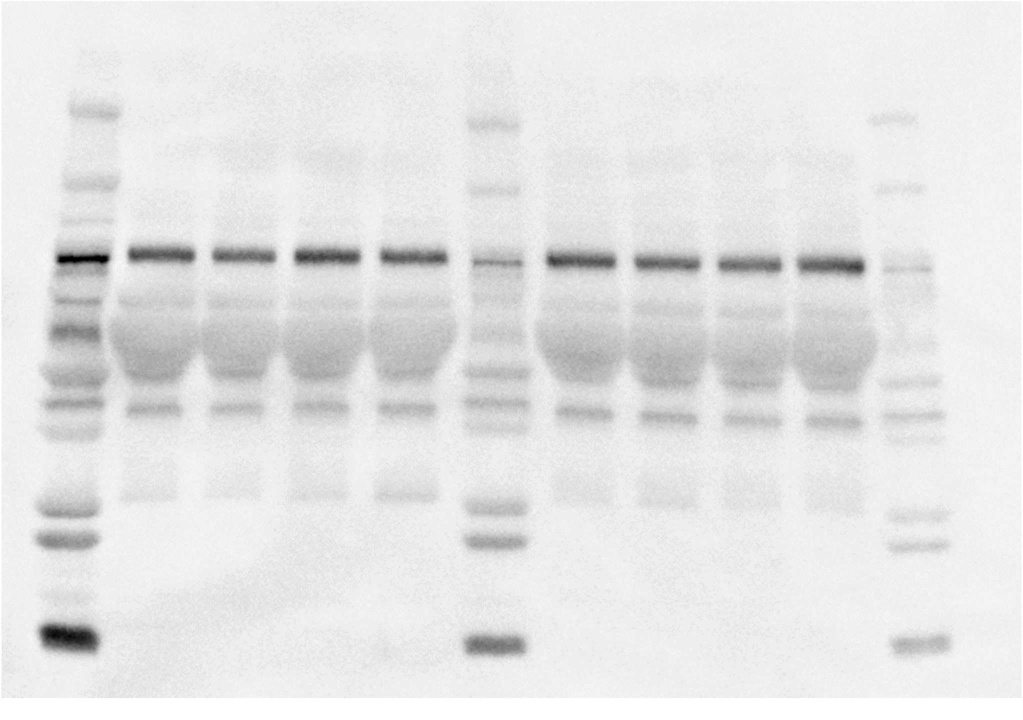

Western Blots (1-4 weeks)

CPT2

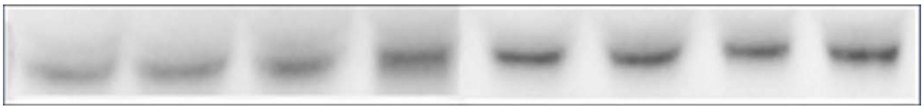

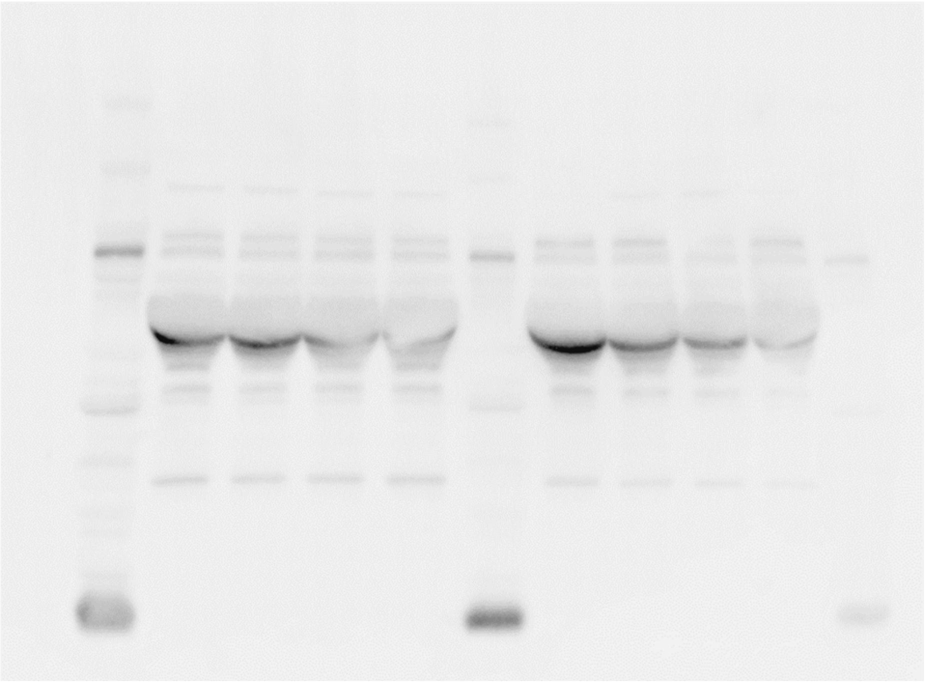

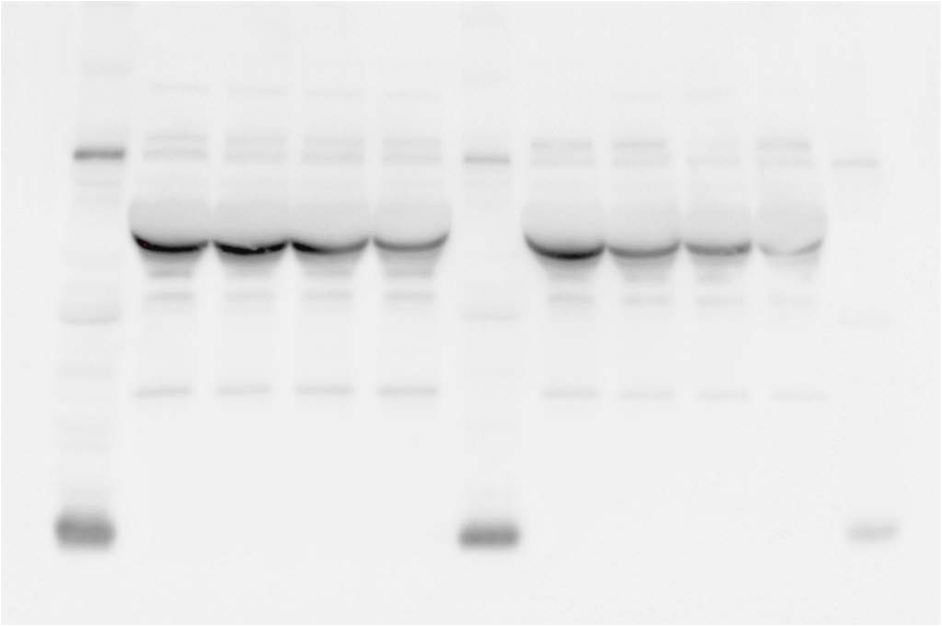

Western Blots (1-4 weeks)

VLCAD

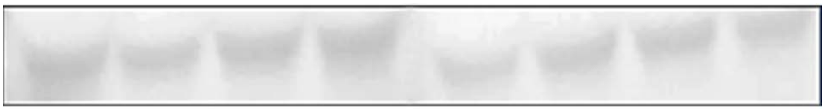

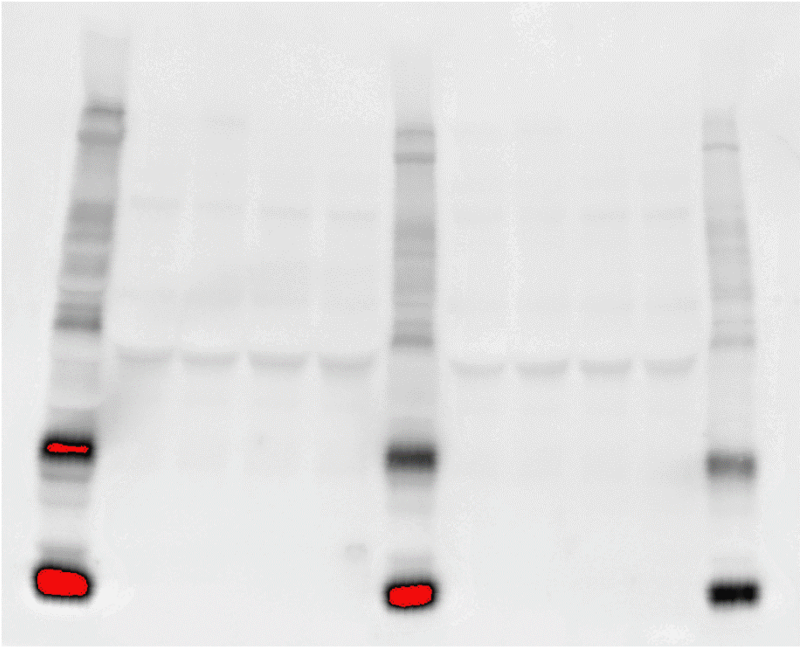

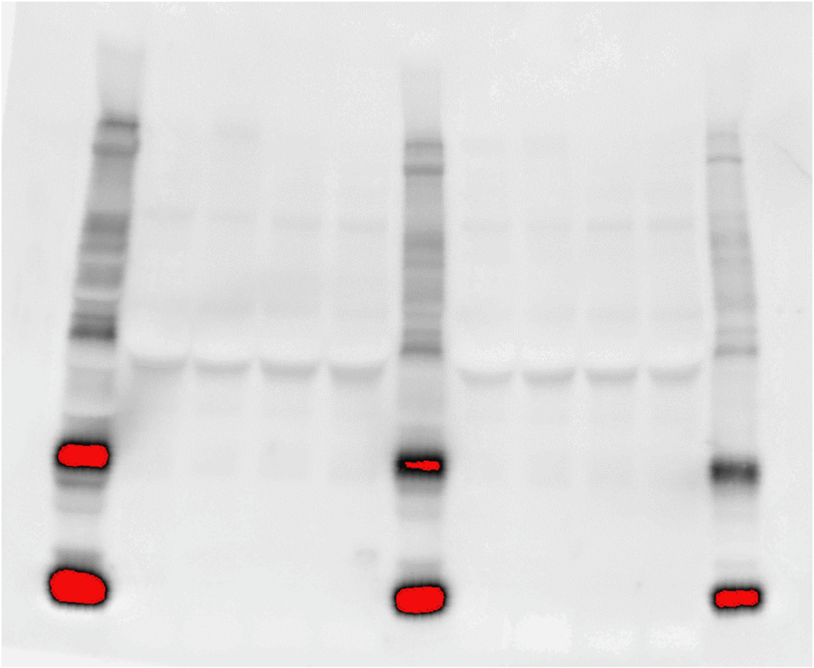

Western Blots (1-4 weeks)

ACOX1

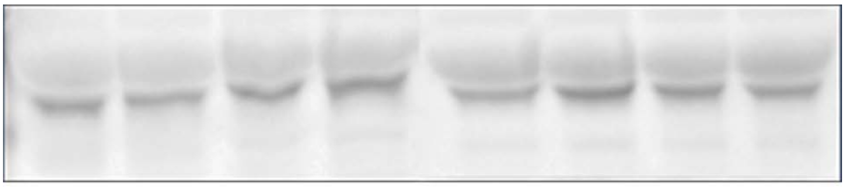

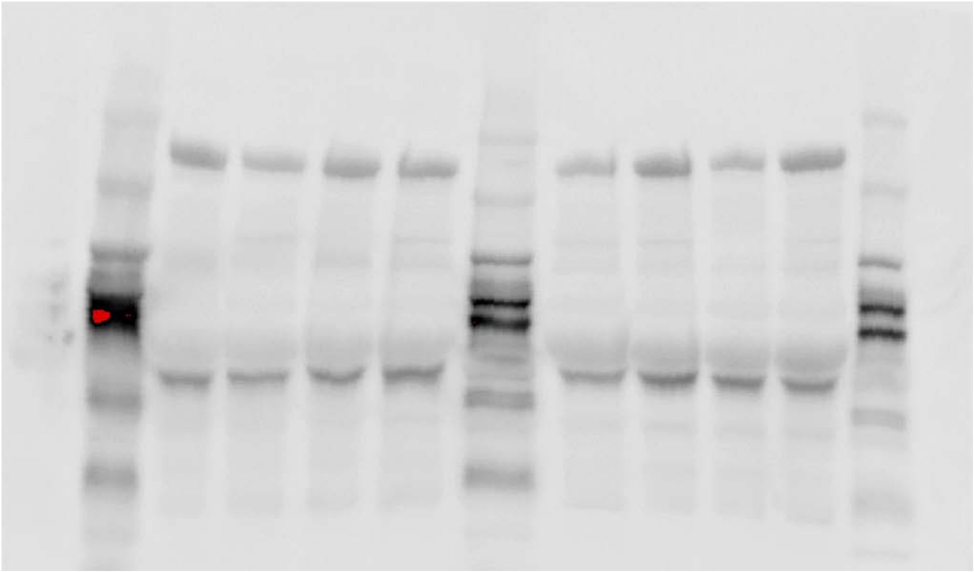

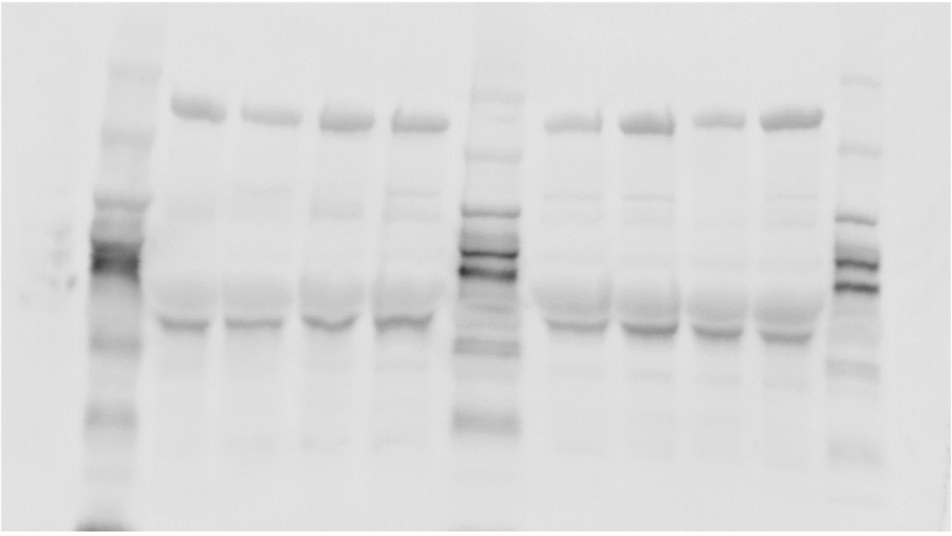

Western Blots (1-4 weeks)

β-Actin

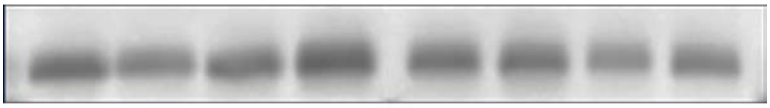

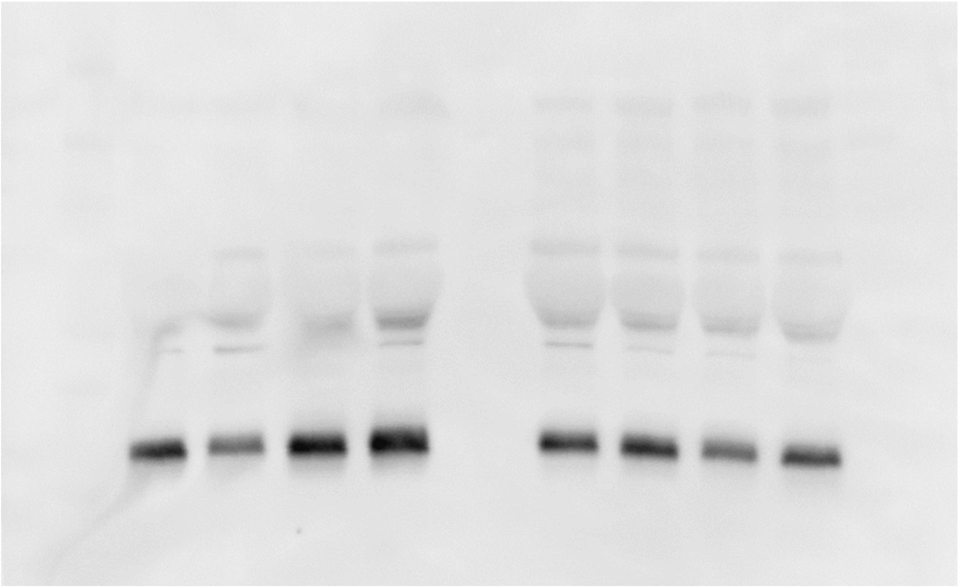

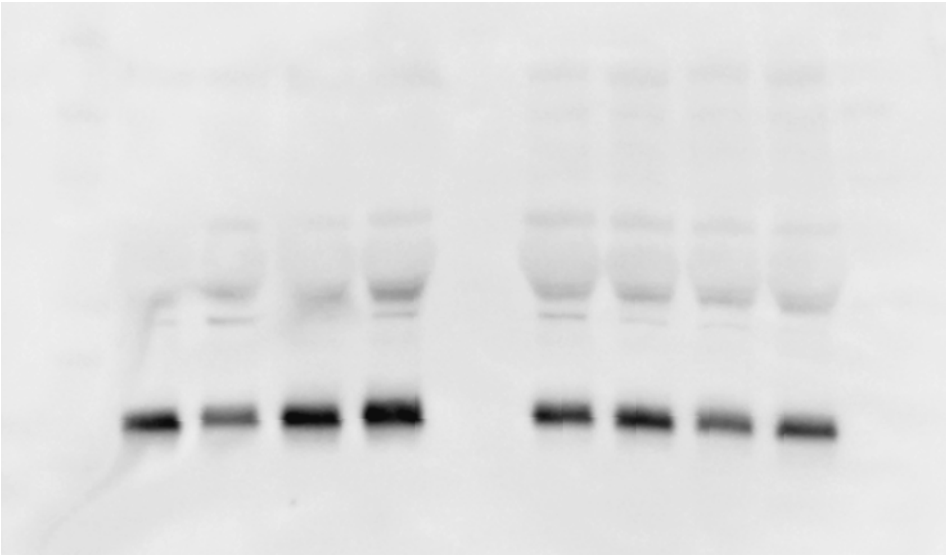

Western Blots (5-8 weeks)

PPARα

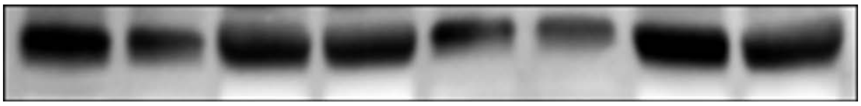

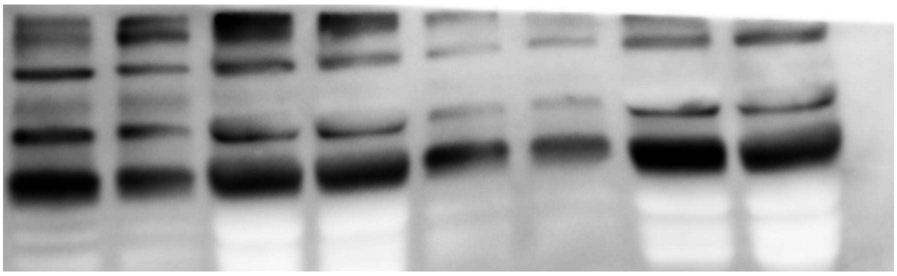

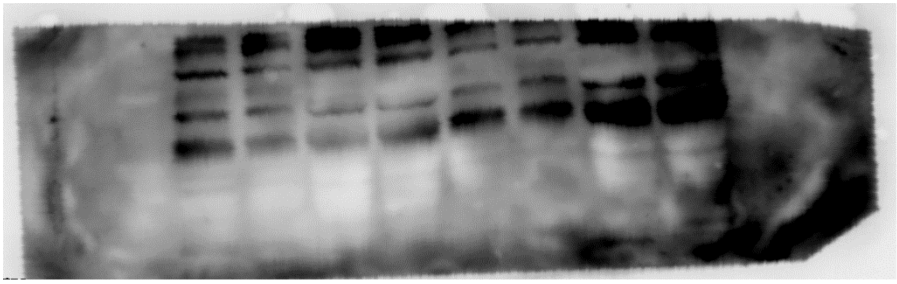

Western Blots (5-8 weeks)

CPT1A

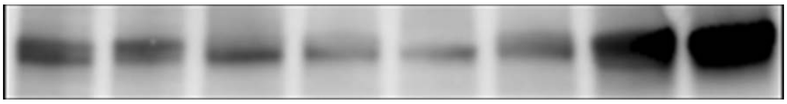

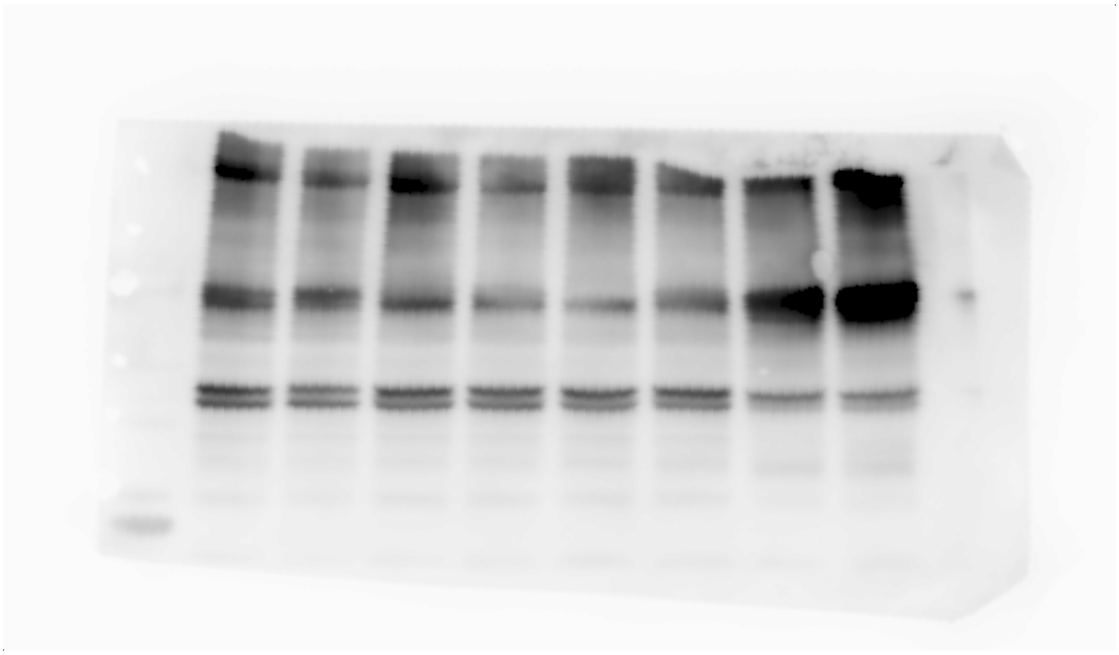

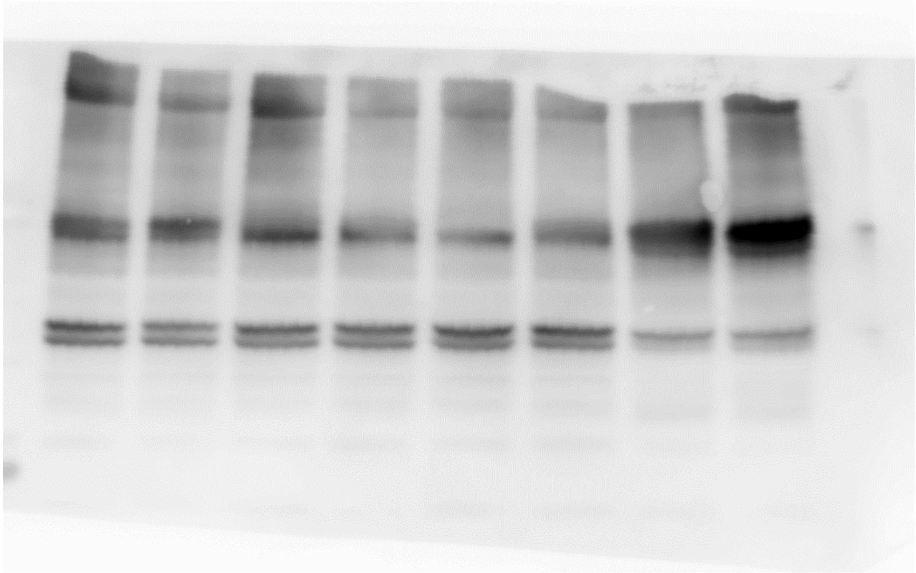

Western Blots (5-8 weeks)

CPT2

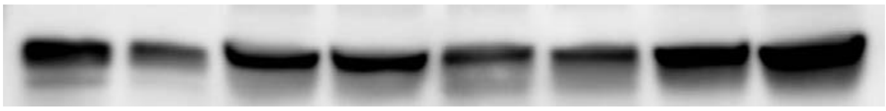

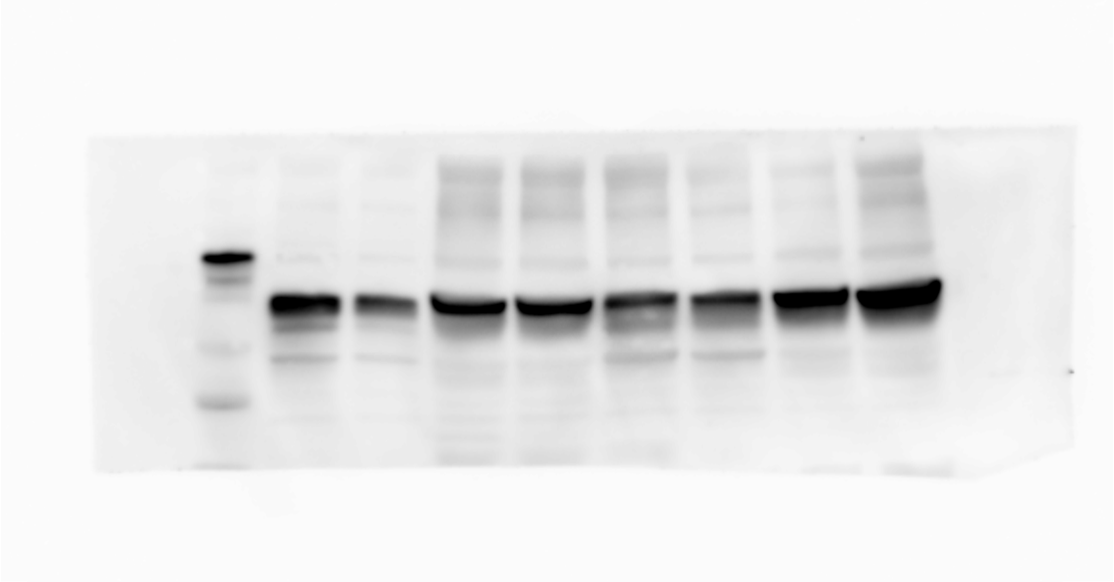

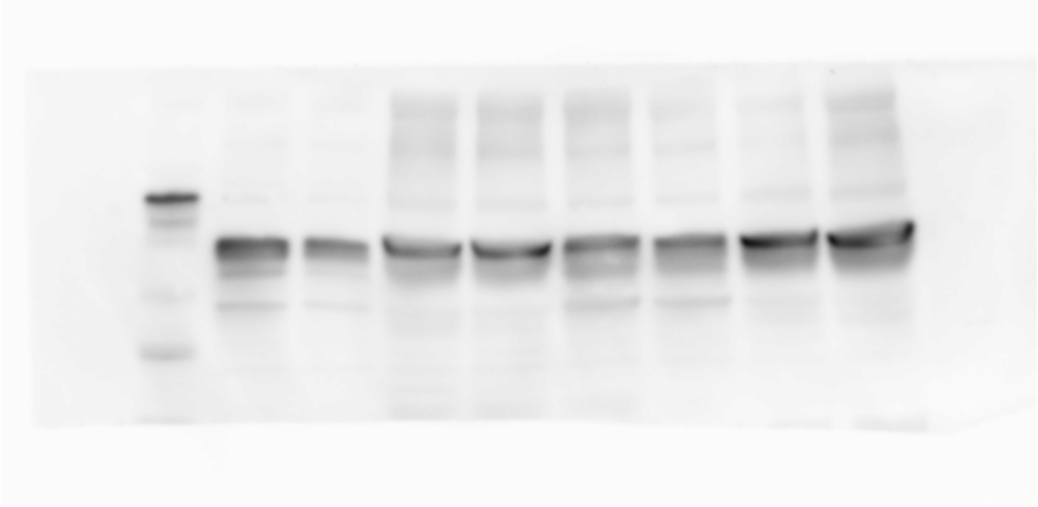

Western Blots (5-8 weeks)

VLCAD

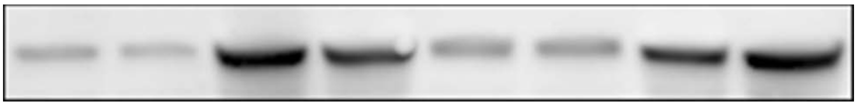

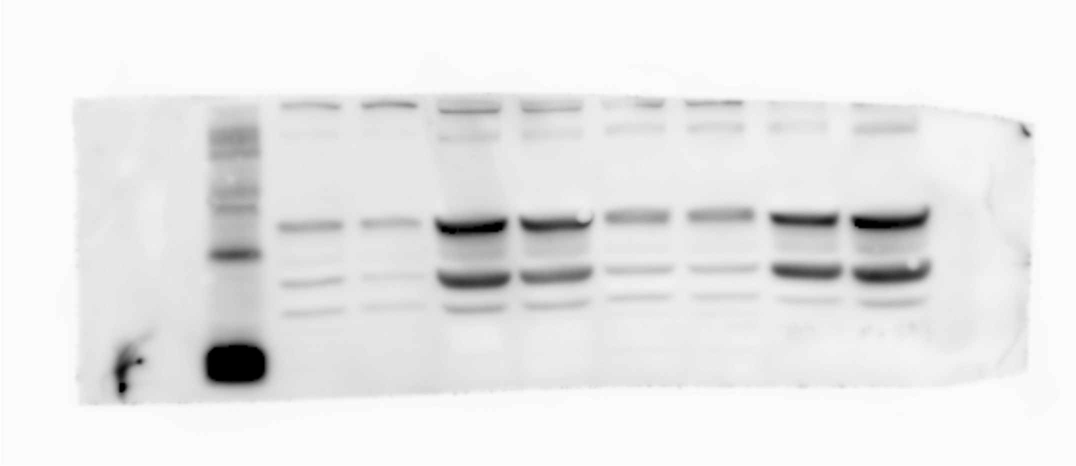

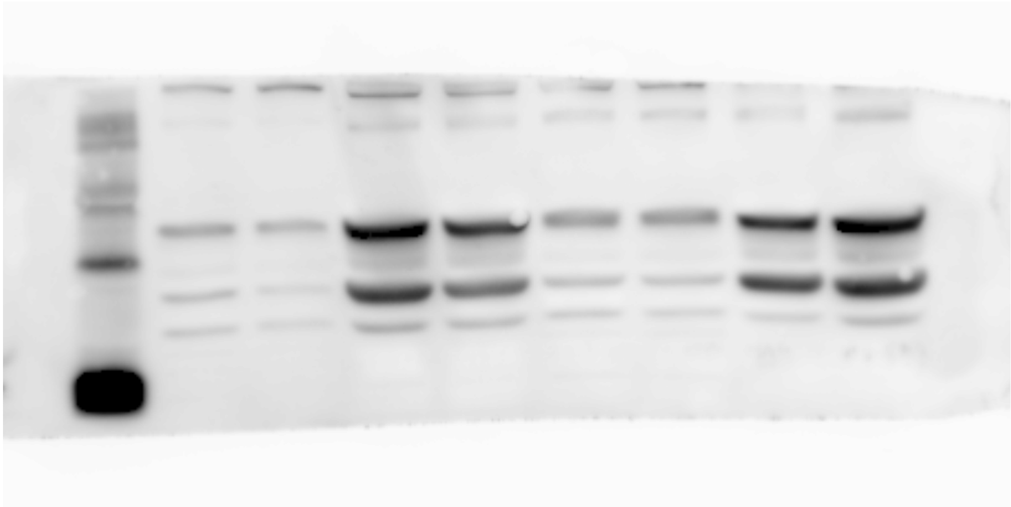

Western Blots (5-8 weeks)

ACOX1

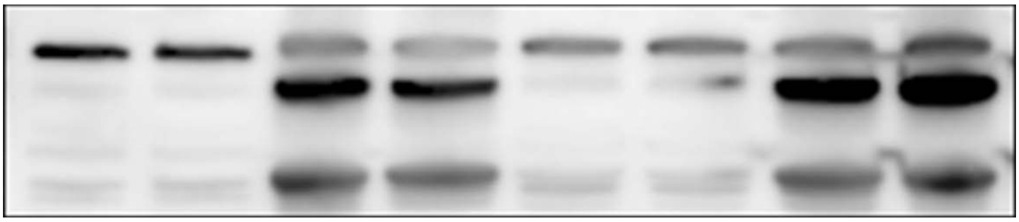

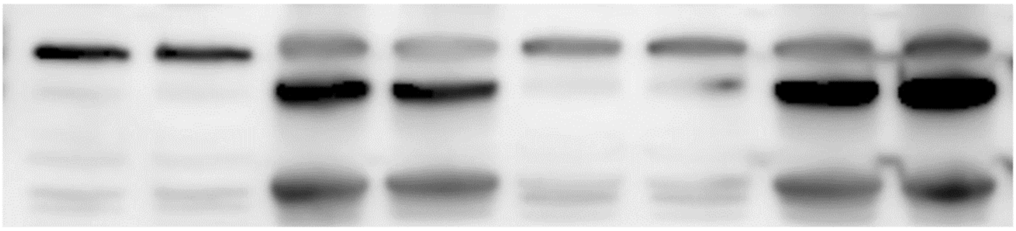

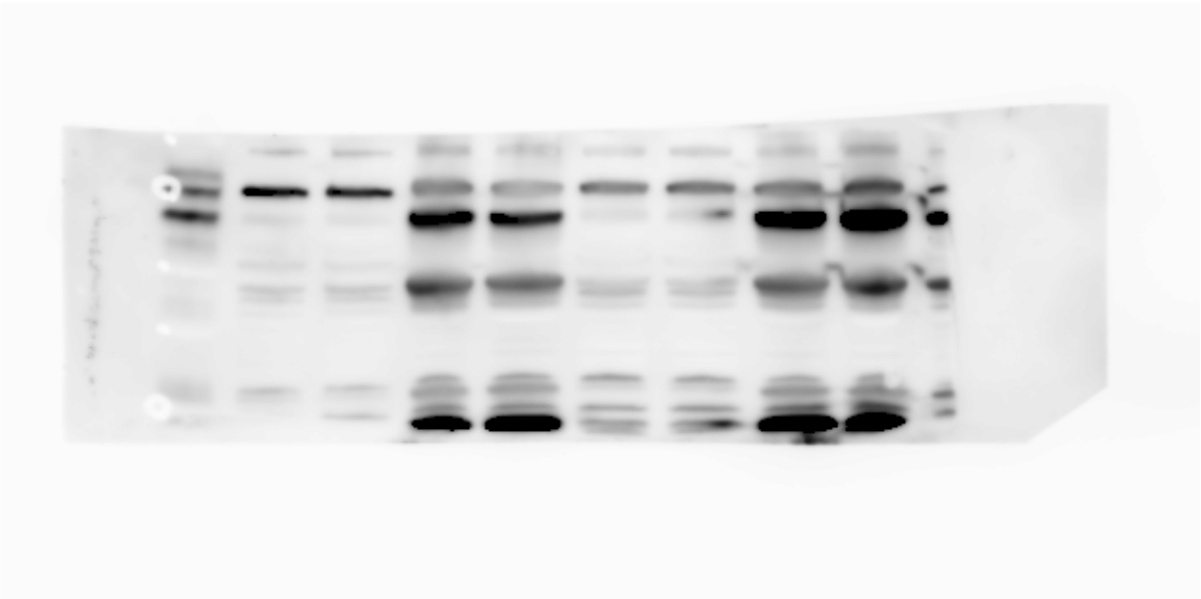

Western Blots (5-8 weeks)

β-Actin

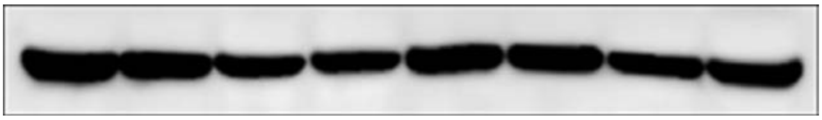

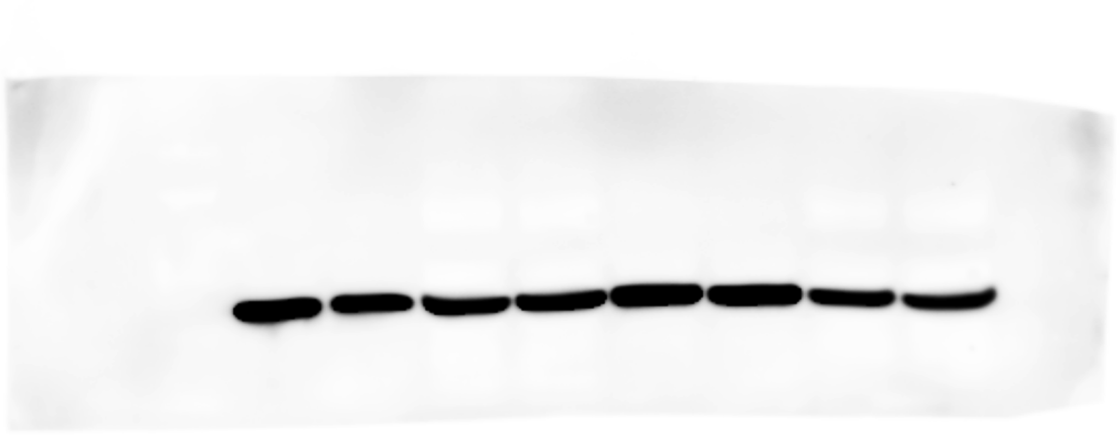

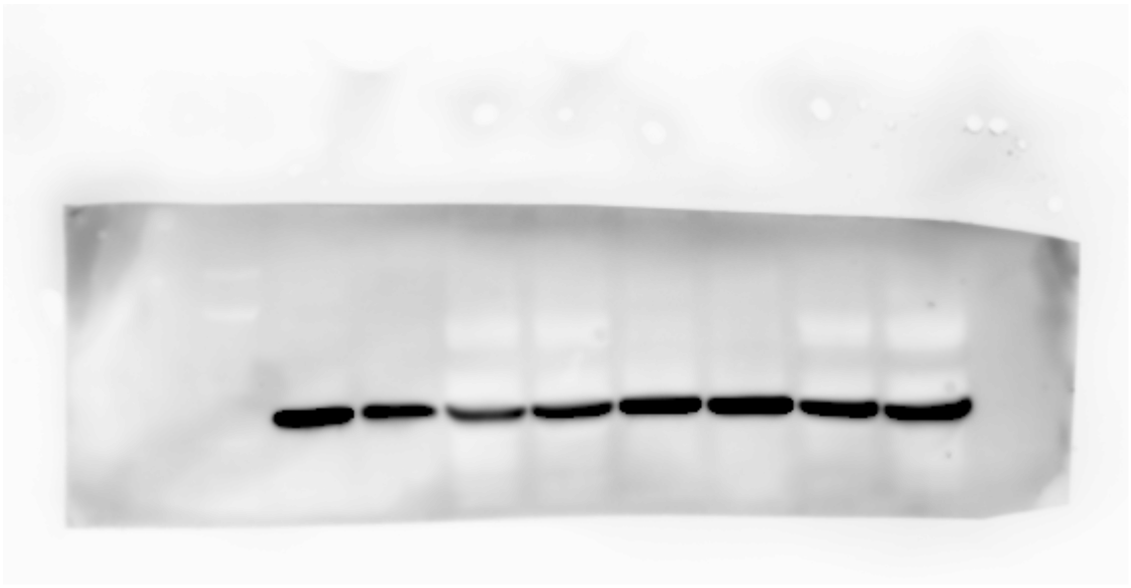

Supplement: Supplementary file 1 — Supplementary Material 1. [file 10020_2025_1210_MOESM1_ESM.pdf]
